# Predicting the evolutionary and functional landscapes of viruses with a unified nucleotide-protein language model: LucaVirus

**DOI:** 10.1093/nsr/nwag376

**Published:** 2026-06-17

**Authors:** Yuan-Fei Pan, Yong He, Yu-Qi Liu, Yong-Tao Shan, Shu-Ning Liu, Jia-Hao Ma, Xue Liu, Xiaoyun Pan, Yinqi Bai, Zan Xu, Tingjun Hou, Zheng Wang, Jieping Ye, Jianguo He, Edward C Holmes, Bo Li, Yao-Qing Chen, Zhao-Rong Li, Mang Shi

**Affiliations:** State Key Laboratory of Wetland Conservation and Restoration, National Observations and Research Station for Wetland Ecosystems of the Yangtze Estuary, Ministry of Education Key Laboratory for Biodiversity Science and Ecological Engineering, and Institute of Eco-Chongming, School of Life Sciences, Fudan University, Shanghai 200433, China; National Key Laboratory of Intelligent Tracking and Forecasting for Infectious Diseases, School of Medicine, Shenzhen Campus of Sun Yat-sen University, Sun Yat-sen University, Shenzhen 518107, China; State Key Laboratory for Biocontrol, School of Medicine, Shenzhen Campus of Sun Yat-sen University, Sun Yat-sen University, Shenzhen 518107, China; Apsara Lab, Alibaba Cloud Intelligence, Alibaba Group, Hangzhou 310030, China; Hangzhou Institute for Advanced Study, University of Chinese Academy of Sciences, Hangzhou 310024, China; HIM-BGI Omics Center, Hangzhou Institute of Medicine (HIM), Chinese Academy of Sciences, Hangzhou 310000, China; BGI Research, Hangzhou 310030, China; Zhejiang Lab, Hangzhou 311121, China; Zhejiang Lab, Hangzhou 311121, China; State Key Laboratory of Biocontrol, School of Life Science, Sun Yat-sen University, Guangzhou 510275, China; School of Public Health (Shenzhen), Shenzhen Campus of Sun Yat-sen University, Sun Yat-sen University, Shenzhen 518107, China; National Key Laboratory of Intelligent Tracking and Forecasting for Infectious Diseases, School of Medicine, Shenzhen Campus of Sun Yat-sen University, Sun Yat-sen University, Shenzhen 518107, China; State Key Laboratory for Biocontrol, School of Medicine, Shenzhen Campus of Sun Yat-sen University, Sun Yat-sen University, Shenzhen 518107, China; National Key Laboratory of Intelligent Tracking and Forecasting for Infectious Diseases, School of Medicine, Shenzhen Campus of Sun Yat-sen University, Sun Yat-sen University, Shenzhen 518107, China; State Key Laboratory of Wetland Conservation and Restoration, National Observations and Research Station for Wetland Ecosystems of the Yangtze Estuary, Ministry of Education Key Laboratory for Biodiversity Science and Ecological Engineering, and Institute of Eco-Chongming, School of Life Sciences, Fudan University, Shanghai 200433, China; BGI Research, Sanya 572025, China; Apsara Lab, Alibaba Cloud Intelligence, Alibaba Group, Hangzhou 310030, China; College of Pharmaceutical Sciences, Zhejiang University, Hangzhou 310058, Zhejiang, China; Apsara Lab, Alibaba Cloud Intelligence, Alibaba Group, Hangzhou 310030, China; Apsara Lab, Alibaba Cloud Intelligence, Alibaba Group, Hangzhou 310030, China; State Key Laboratory of Biocontrol, School of Life Science, Sun Yat-sen University, Guangzhou 510275, China; State Key Laboratory of Biocontrol/Southern Laboratory of Ocean Science and Engineering (Guangdong, Zhuhai), School of Marine Sciences, Sun Yat-sen University; Guangzhou 510275, China; School of Medical Sciences, The University of Sydney, Sydney, NSW 2006, Australia; State Key Laboratory of Wetland Conservation and Restoration, National Observations and Research Station for Wetland Ecosystems of the Yangtze Estuary, Ministry of Education Key Laboratory for Biodiversity Science and Ecological Engineering, and Institute of Eco-Chongming, School of Life Sciences, Fudan University, Shanghai 200433, China; State Key Laboratory for Vegetation Structure, Functions and Construction (VegLab), Ministry of Education Key Laboratory for Transboundary Ecosecurity of Southwest China, Institute of Biodiversity, School of Ecology and Environmental Science, and Southwest United Graduate School, Yunnan University, Kunming 650500, China; School of Public Health (Shenzhen), Shenzhen Campus of Sun Yat-sen University, Sun Yat-sen University, Shenzhen 518107, China; Apsara Lab, Alibaba Cloud Intelligence, Alibaba Group, Hangzhou 310030, China; Zhejiang Lab, Hangzhou 311121, China; National Key Laboratory of Intelligent Tracking and Forecasting for Infectious Diseases, School of Medicine, Shenzhen Campus of Sun Yat-sen University, Sun Yat-sen University, Shenzhen 518107, China; State Key Laboratory for Biocontrol, School of Medicine, Shenzhen Campus of Sun Yat-sen University, Sun Yat-sen University, Shenzhen 518107, China; Guangdong Provincial Center for Disease Control and Prevention, Guangzhou 511430, China

**Keywords:** virology, artificial intelligence, emerging diseases, foundation model, virus evolution, pandemic preparedness

## Abstract

Predicting viral evolution and function remains a central challenge in biology, hindered by high sequence divergence and limited knowledge compared to cellular organisms. Here, we introduce LucaVirus, a multi-modal foundation model for viruses, trained on 25.4 billion nucleotide and amino acid tokens covering a vast majority of catalogued viral diversity. LucaVirus learns biologically meaningful representations that reflect relationships between sequences, protein/gene homology, and evolutionary divergence. Using these embeddings, we developed downstream models that address key virology tasks: identifying hidden viruses in genomic ‘dark matter’, annotating enzymatic activities of uncharacterized proteins, predicting viral evolvability, and identifying antibody candidates for emerging viruses. LucaVirus demonstrates competitive performance in three tasks and matches leading models in the fourth with one-third the parameters. Together, these findings demonstrate the utility of a unified foundation model in analyzing viral sequence data and establish LucaVirus as an efficient and versatile platform for AI-driven virology, from virus discovery to functional and therapeutic predictions.

## INTRODUCTION

The global expansion of metagenomic sequencing has revealed a vast and rapidly growing diversity of viruses across animals [1], plants [2], and a wide range of environments [3,4]. Despite their often-small genomes, viruses encode nearly all biologically relevant information required for host interaction [5], immune evasion [6], and transmission [7]—making viral genomes and proteomes compact yet comprehensive blueprint of life. However, translating this genomic wealth into biological insight remains challenging [8]. This bottleneck is mainly driven by the high evolutionary rates of viruses [9], which leads to high levels of extreme sequence divergence [10], particularly among RNA viruses. Consequently, conventional bioinformatic methods based on sequence homology and conserved domain detection often lack the sensitivity and precision required to bridge the gap between viral discovery, functional characterization, and public health applications.

Recent advances in artificial intelligence, particularly large language models (LLMs), offer new opportunities to learn complex biological representations directly from sequence data. Models such as the ESM series [11,12] and the Evo series

[13,14] have demonstrated capabilities in protein function/structure prediction and *de novo* generation across cellular organisms. However, their application to virology has been constrained by a unique intersection of technical and biosafety challenges. First, viral sequences are substantially underrepresented in generalist foundation models trained predominantly on cellular organisms. Viral genomes exhibit distinct evolutionary constraints and composition biases that differ fundamentally from those in cellular life [15], including high coding density and widespread use of non-canonical mechanisms such as polyprotein processing and programmed ribosomal frameshifting (PRF) [16,17], features that are underrepresented in the data sets of existing models. Second, most existing models are unimodal, focusing exclusively on either protein or nucleotide sequences. Viral genomes, however, encode rich evolutionary information [18,19]—such as codon usage biases indicative of host range—that is not explicitly captured in amino acid sequences. Conversely, proteins often preserve functional conservation better than rapidly evolving nucleotides, making protein-level representations valuable for detecting remote homology. Finally, the rise of generative AI has introduced a biosecurity paradox: while models like Evo possess the architecture to learn genomic grammar, concerns regarding the potential synthesis of hazardous pathogens have led to the intentional exclusion or masking of viral sequences from their training corpora. This safety-driven blind spot leaves the virology community without high-capacity foundation models tailored to the diverse characteristics of the virosphere.

To address these challenges, we present LucaVirus, a unified genome–protein language model for viruses. Initialized from the general-purpose foundation model LucaOne [20], LucaVirus employs transfer learning followed by pre-training on one of the most comprehensive viral sequence collections assembled to date. By jointly modeling nucleotide and amino acid sequences, LucaVirus captures the unique grammatical and semantic dependencies underlying viral evolution, a design validated by rigorous ablation analyses. This unified framework supports diverse downstream applications, including discovery of highly divergent viruses, functional annotation, fitness landscape modeling, and antibody–antigen interaction prediction. Together, LucaVirus offers a generalizable and interpretable AI framework for advancing both basic virological research and pandemic preparedness.

## RESULTS

### LucaVirus: a unified model for viral genomes and proteins

**Figure 1.**
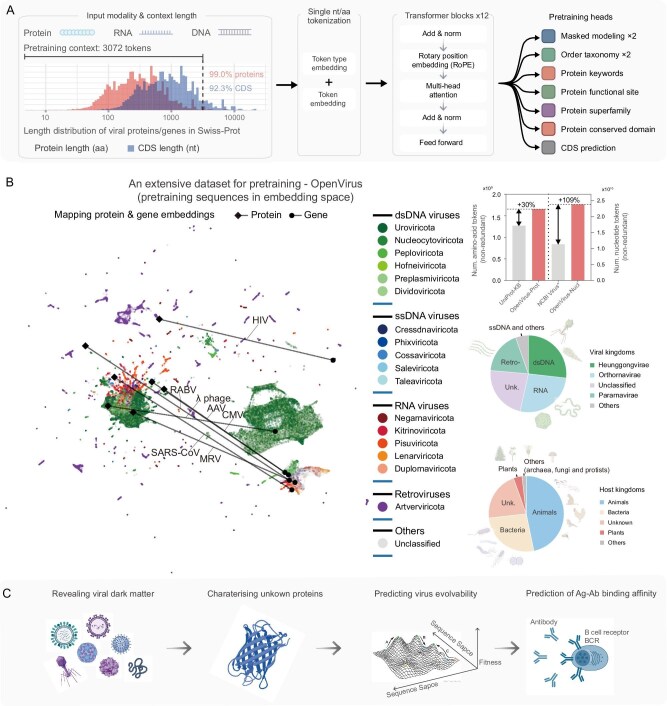
LucaVirus: a unified multi-modal foundation model for the global virosphere. (A) Architecture and pretraining tasks of LucaVirus. (B) Overview of the OpenVirus pretraining data set. Left: visualization of pretraining protein and nucleotide sequences in LucaVirus embedding space, with color indicating taxonomy and lines indicating the mapping between corresponding protein and nucleotide sequences. Right, bar plot: total tokens of non-redundant protein and nucleotide sequences in our pre-training data set compared with mainstream public databases—UniProtKB and NCBI Virus. Right, pie plot: taxonomic distribution and host diversity within the training corpus. (C) Schematic overview of the four downstream applications enabled by LucaVirus.

LucaVirus is a unified genome–protein language model with one billion parameters, designed to interpret viral sequence at single-nucleotide and single-amino acid resolution (Fig. 1A). With the rapid expansion of viral diversity, there is a growing requirement for a comprehensive and scalable model capable of capturing both genetic and functional characteristics across a broad range of viral taxa. LucaVirus addresses this need by integrating a vast majority of catalogued viral sequences into a single, cohesive framework.

To support such broad representation, we constructed OpenVirus, a large-scale pre-training corpus comprising 15.7 million viral sequences, including 10.4 million nucleotide and 5.2 million protein sequences, totaling 25.4 billion tokens (Fig. 1B). This data set substantially exceeds existing repositories, containing nearly twice the number of non-redundant tokens found in NCBI virus and 30% more than UniProt [21]. Importantly, OpenVirus incorporates recent large-scale viral discovery efforts [10,22–26], contributing 1663 163 previously uncatalogued ‘species-level’ taxa (80% ANI) absent from public databases (Table S1). The data set spans all major viral realms—including double-stranded DNA viruses (*Heunggongvirae*, 27%), RNA viruses (*Orthornavirae*, 26%), and retroviruses (*Pararnavirae*, 20%), while covering viruses infecting hosts across all six cellular kingdoms (Fig. 1B).

To effectively learn from this diverse data and to prioritize sequence understanding over generation in light of biosecurity considerations, LucaVirus employs a customized encoder-only Transformer architecture with 1 billion parameters. The model was initialized with weights from LucaOne [20], a general-purpose biological foundation model. This transfer learning strategy allows LucaVirus to inherit fundamental biological sequence priors before extensive virus-specific training. To balance efficiency and expressiveness, we reduced the model to 12 transformer layers while expanding the context window to 3072 tokens. This extended window is critical for viral genomics, allowing LucaVirus to capture long-range dependencies across 99% of viral proteins and 92% of coding sequences (CDS) in SwissProt [21] (Fig. 1A). Furthermore, LucaVirus was trained using a multi-task semi-supervised objective [27,28] rather than standard masked language modeling (MLM), explicitly enforcing sequence–function associations and enhancing the model’s ability to decode viral functional semantics.

### Ablation studies evaluate the performance of a unified multi-modal design

To justify our architectural design, we benchmarked the standard LucaVirus against three variants: protein-only (LucaVirus-Prot), nucleotide-only (LucaVirus-Nucl), and a model trained exclusively on the MLM objective without biological supervision (LucaVirus-Mask). Across nine downstream tasks, the unified multi-modal LucaVirus outperformed all variants in seven, suggesting that integrating nucleotide and protein representations is essential for a comprehensive understanding of viral biology (Fig. S2; Table S3). Although LucaVirus-Prot showed marginal advantages in capsid and enzyme prediction (by 0.02% and 0.22%, respectively), these gains were task-specific and did not generalize. And LucaVirus-Mask consistently underperformed across benchmarks, highlighting the limitations of purely syntactic language modeling and underscoring the importance of biologically informed training objectives for learning functional viral representations.

To further assess model stability and rule out ‘catastrophic forgetting’ [29], we re-evaluated LucaVirus on ten general biological benchmarks originally developed for the LucaOne model [20]. LucaVirus achieved comparable performance on 90% of these tasks (Fig. S3; Table S4), confirming that our transfer learning strategy effectively specializes the model for the virosphere while preserving fundamental biological syntax and generalization capacity.

### LucaVirus recovers fundamental genomic syntax and non-canonical translation logic from raw sequence

LucaVirus maps viral sequences into a biologically structured, high-dimensional latent space without explicit supervision. Nucleotide-level embeddings capture features consistent with the fundamental rules of the central dogma, resolving multiple layers of genomic organization, including base identity, coding versus non-coding regions, codon phase structure, and synonymous codons (Fig. S4). This latent organization was quantitatively validated across all four features via permutational multivariate analysis of variant (PERMANOVA, 999 permutations, *P* < 0.05; Table S5), confirming that these biologically meaningful dimensions emerge intrinsically from model training.

To assess the linear separability of these representations, we conducted systematic linear probing benchmarks against state-of-the-art genomic language models (Fig. S5; Table S6) LucaVirus achieved 86%–100% accuracy across five core genomic discrimination tasks, including CDS identification, base identity, codon phase, amino acid identity, and codon identity. This level of performance was matched only by LucaOne (66%–100%). Notably, LucaVirus reached 93.8% accuracy in CDS identification, achieving higher accuracy than several existing genome language models such as Evo-1-8k-base (62.9%) [14], Evo2-7B (74.3%) [13], and Nucleotide Transformer-v2-500 M (74.7%) [30].

Beyond standard syntax, LucaVirus captures subtle, virus-enriched genomic features that are typically difficult to model. The embeddings accurately reconstruct relative nucleotide positions within codons (Fig. S6) and preserve phase consistency across PRF boundaries. Quantitative evaluation using the FSDB database [31] (Fig. S7; Table S6) demonstrated that LucaVirus distinguishes codon phases with 98% precision, This serves as a proof-of-principle demonstration of the model’s potential to decode non-canonical translational rules; however, we acknowledge that this validation is currently limited to established motifs, and its robust application to uncharacterized viral genomes remains to be further established.

### Embedding geometry encodes evolutionary distance and enables embedding-based sequence alignment

**Figure 2.**
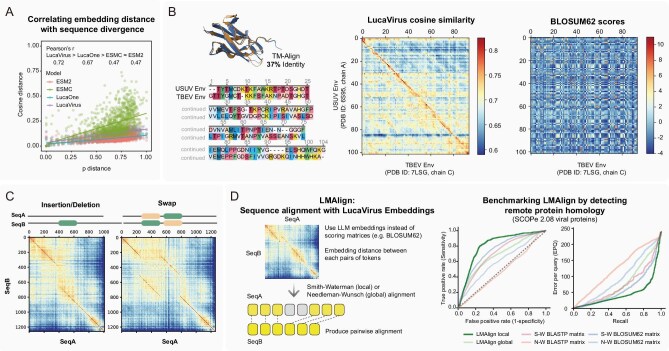
Learned representations by LucaVirus capture evolutionary divergence and enable embedding-based alignment. (A) Correlation between embedding space geometry and genetic distance. The scatter plot compares the Pearson’s *r* of cosine similarity against *p* distance for LucaVirus versus other protein language models (LucaOne, ESMC, ESM2) on unseen viral protein families during LucaVirus pretraining. (B) Structural superposition (top) and sequence alignment (bottom) of two remote homologs and comparison of pairwise similarity matrices generated by LucaVirus embeddings versus the BLOSUM62 scoring matrix. (C) Visualization of structural variations in the embedding space. (D) Schematic of LMAlign alignment algorithm and benchmarks on remote viral protein homology detection. ROC curves and error per query (EPQ) plots.

The geometric structure of the LucaVirus embedding space correlates with evolutionary divergence. Across 5566 reference protein families and 938 ‘dark matter’ families, we observed a strong and consistent correlation between embedding cosine similarity and genetic distance measured by *p*-distance (Fig. S8; Table S7). Importantly, this relationship generalizes well to sequences unseen during pre-training (Pearson’s *r* = 0.71; Fig. 2A) and extends robustly to nucleotide sequences (Table S8), demonstrating that LucaVirus embeddings capture fundamental evolutionary signals rather than data-set-specific artifacts.

This representational capacity effectively captures remote homology. For example, in the envelope proteins of Usutu virus and tick-borne encephalitis virus, which share only 37% identity (Fig. 2B). Visualization of pairwise residue scores demonstrate that LucaVirus embeddings distinctively highlight alignable regions, in sharp contrast to the noisy background produced by the standard BLOSUM62 scoring matrix [32] (Fig. 2B). The embeddings further exhibit sensitivity to structural variations such as indels and domain rearrangements (Fig. 2C), indicating that contextual and structural constraints are implicitly encoded in the latent space. Building on these insights, we developed LMAlign, a novel sequence embedding-based sequence alignment algorithm that leverages LucaVirus representations to guide alignment (Fig. 2D; Fig. S9). Benchmarking on the SCOPe 2.08 data set [33] confirmed that LMAlign achieves higher sensitivity than classic Smith–Waterman and Needleman–Wunsch algorithms in detecting remote structural homology (Fig. 2D).

### Evaluating LucaVirus across virological tasks

Decoding the global virosphere requires overcoming a series of interconnected barriers that span from virus discovery to pandemic preparedness. Beyond the fundamental challenge of identifying highly divergent viruses hidden within genomic dark matter, researchers face the critical bottleneck of assigning biological functions to the vast majority of viral proteins that lack cellular homologs. Furthermore, effective viral surveillance demands not only static characterization but also the dynamic forecasting of viral fitness landscapes and the rapid identification of neutralizing antibodies. To demonstrate the versatility of LucaVirus, we systematically assessed its performance across these four pivotal domains, utilizing lightweight downstream models to interpret the rich biological semantics encoded in its pre-trained embeddings.

For each task, we employed lightweight, task-specific downstream models that operate directly on the embeddings generated by pre-trained language models (see Methods). These downstream models share a unified, minimal architecture comprising a single attention-based pooling layer, a fully connected layer, and a task-specific prediction head (Fig. S10), while keeping the upstream language model frozen. This design minimizes trainable parameters, improves computational efficiency, and reduces dependence on large-scale, labeled data sets—a major advantage in virology, where experimentally validated data are scarce.

### Challenge 1: High-sensitivity discovery of viral hallmark proteins from genomic dark matter

**Figure 3.**
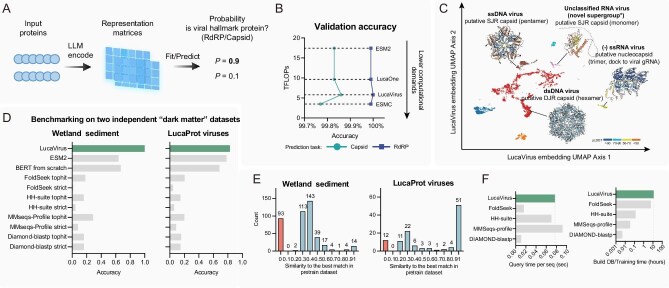
Uncovering viral dark matter through efficient hallmark protein identification. (A) Workflow for the binary classification of viral hallmark proteins (RdRP and Capsid) using LucaVirus embeddings. (B) Comparison of the accuracy and computational complexity (floating point operations per inference, TFLOPs) among three LLMs in RdRP and capsid protein prediction. (C) UMAP visualization of the LucaVirus embedding space for viral capsid proteins. (D) Performance on two independent ‘dark matter’ test sets of divergent viral proteins. (E) Distribution of sequence identity between the ‘dark matter’ test set and the nearest matches in the pre-training data set. (F) Efficiency comparison for query time per sequence and database construction/training time.

Building on our previous work [10], we targeted two hallmark viral proteins—the RNA-dependent RNA polymerase (RdRP) and viral capsid proteins—to uncover unclassified RNA viruses present within genomic dark matter (Fig. 3A). RdRP is a highly conserved enzyme critical for RNA virus replication and rarely found in cellular organisms [34], making it a robust marker for virus discovery [35]. In contrast, viral capsid proteins are structurally essential but highly divergent, posing a major challenge for conventional homology-based detection [36].

On a validation set comprising 23 000 RdRPs and 48 000 capsid proteins, the LucaVirus hallmark protein identification model achieved accuracies of 100% for RdRP and 99.86% for capsids (Fig. 3B; Tables S6 and S7). Notably, LucaVirus matched the performance of the substantially larger ESM2-3B model while reducing reference cost from 17 TFLOPs to 5 TFLOPs per inference, representing a 3.4-fold increase in efficiency. Through visualization, we showed that LucaVirus robustly generalizes across diverse capsid architectures, including nucleocapsids, single jelly roll, and double jelly roll folds, despite substantial sequence and structural heterogeneity.

To rigorously evaluate real-world generalization, we benchmarked LucaVirus on a curated set of 115 structurally validated proteins from recently discovered RNA viruses (68 capsids and 47 non-capsids; Extended Data Set 1) [10], after excluding any sequences with any significant hit to capsid training data set or sharing >50% identity with the pre-training data. Under these stringent conditions, LucaVirus achieved 100% recall and accuracy (Fig. 3D; Table S9). In contrast, ESM2-3B reached only 66% accuracy (65.3% recall), while structure-based (FoldSeek) [37] and sequence- or profile-based methods (DIAMOND blastp [38], HHblits [39], and MMseqs2 [40]) showed accuracies below 22.6% and recalls below 10% (Fig. 3D). To further assess the model’s generalizability, we applied LucaVirus to a newly derived data set derived from wetland sediments. Crucially, this data set was absent from the OpenVirus corpus used for model training; its average sequence identity to the OpenVirus corpus was even lower than that of the previous test set (Fig. 3E). For this data set, 430 viral capsid proteins validated through either structural confirmation or virus-associated evidence (e.g. co-localized viral hallmark genes, capsid-specific domains hits, or NCBI NR taxonomic classification) served as the benchmark gold standard. On this data set, LucaVirus consistently outperformed all other tested AI-based and bioinformatics methods (Fig. 3D), further supporting the robust generalizability of our approach.

Finally, we assessed scalability on large-scale data sets using identical hardware (128-core, 256-thread CPU, and single NVIDIA A100 GPU). LucaVirus achieved a per-sequence inference time of 0.077 s, positioning it between HHblits and MMseqs2 in speed (Fig. 3F). This inference speed uniquely positions LucaVirus as a method capable of combining high sensitivity with the throughput required for large-scale metagenomic mining. Although model training requires a one-time computational cost comparable to database indexing for FoldSeek (Fig. 3F), LucaVirus thereafter provides an efficient and scalable solution for systematic exploration of viral genomic dark matter.

### Challenge 2: Improving functional annotation coverage of the viral proteome

Although metagenomics has rapidly expanded the known virosphere, functional annotation of viral proteins remains a critical bottleneck due to extreme sequence divergence, which severely limits conventional homology-based methods. We therefore evaluated LucaVirus for viral protein function prediction across increasingly challenging benchmarks.

**Figure 4.**
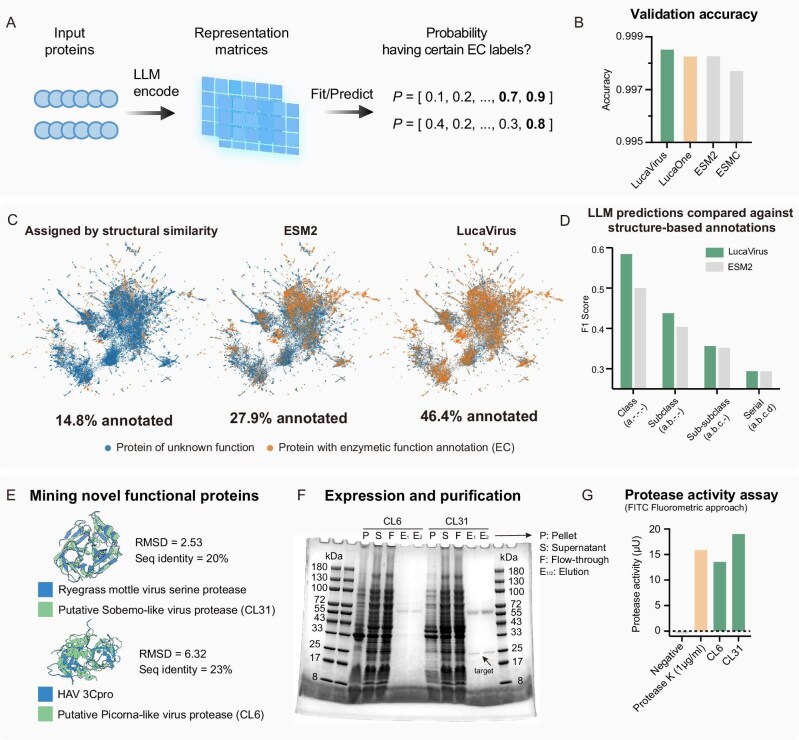
Functional annotation and experimental validation of novel viral enzymes. (A) Schematic of the enzyme function prediction task, mapping input sequences to EC probabilities. (B) Validation accuracy comparison across models. LucaVirus achieves superior accuracy (>0.998) in assigning EC labels to viral proteins. (C) UMAP visualization of the protein embedding space from the Nomburg *et al.* data set [8]. Points are colored by predicted (orange) or unknown (blue) enzymatic function. (D) Performance evaluation against structural ground truth on the Nomburg *et al.* data set [8]. (E) Discovery of two novel viral protease and structure alignment against known protease. (F) SDS-PAGE analysis of the expression and purification of two putative viral proteases (CL6 and CL31) predicted by the model. (G) *In vitro* proteolytic activity assay using a fluorescence-based method.

We first evaluated LucaVirus on a standard viral Enzyme Commission (EC) number prediction task using UniProtKB-derived data sets (Fig. 4A). LucaVirus achieved 99.97% accuracy with an F1 score of 0.9889 (Fig. 4B; Table S10), matching the performance of the much larger ESM2-3B model while operating with substantially fewer parameters, demonstrating its efficiency.

Because standard UniProt benchmarks may be saturated—making it difficult to differentiate model performance—we next evaluated LucaVirus on a rigorously curated, structure-based data set [8] in which viral proteins were annotated through structural alignment. While the original structural approach annotated only 14.8% of the proteins and the ESM2-based model reached 27.9%, LucaVirus expanded annotation coverage to 46.4% (Fig. 4C). When benchmarked against structural ground truth—excluding sequences with >50% identity to pretraining data—LucaVirus achieved a recall of 44.5% and an F1 score of 0.587 at the enzyme class level, outperforming ESM2-3B (recall: 35.1%, F1:0.5; Table S10).

To test real-world discovery potential, we applied LucaVirus to identify putative novel proteases (EC 3.4.-.-) from the genomic ‘dark matter’ of recently discovered RNA viruses [10]. LucaVirus identified 8074 candidate proteins (Extended Data Set 2), from which 46 clusters were selected for structural validation using ColabFold [41] (Extended Data Set 3). Eight clusters displayed clear structural similarity to known proteases (Foldseek *E*-value < 10^−5^), despite sharing only ∼12% sequence identity, well within the homology ‘twilight zone’. Two candidates (CL31 and CL6) were experimentally validated: both were successfully expressed using an *Escherichia coli* system, showed oligomerization consistent with viral proteases, and exhibited protease activity *in vitro* (Fig. 4G). These discoveries were achieved using sequence data alone without structural inputs, which underscores LucaVirus’s ability to infer deep sequence-to-function relationships in cases where conventional alignment- and structure-based methods show limited sensitivity.

### Challenge 3: Predicting the viral fitness landscapes and evolutionary potential

**Figure 5.**
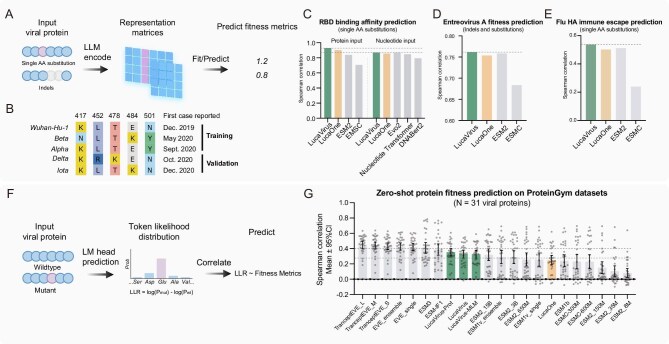
Forecasting viral evolvability and fitness landscapes via zero-shot inference. (A) Workflow for predicting fitness effects of single amino acid substitutions and indels in using a supervised approach. (B) The temporal split of SARS-CoV-2 variants used for training and validation. (C–E) Spearman correlation coefficients between model predictions and experimental DMS data for SARS-CoV-2 RBD binding affinity (C), enterovirus A fitness (D), and HA immune escape (E). (F) Schematic of the zero-shot prediction strategy using the log-likelihood ratio (LLR) of mutant versus wildtype tokens. (G) Zero-shot performance benchmark on the ProteinGym data set. The scatter plot shows the mean Spearman correlation (with 95% CI) for LucaVirus compared to state-of-the-art models.

Accurately modeling viral fitness landscape is essential for predicting viral evolvability. Unlike cross-species functional annotation, this task requires resolving subtle fitness effects of intra-species variation. We evaluated LucaVirus using deep mutational scanning (DMS) data, which systematically quantify the fitness impact of single mutations [42]. Focusing first on receptor-binding affinity of SARS-CoV-2 Spike RBD variants (Fig. 5A and B; Table S11), LucaVirus achieved state-of-the-art performance with a Spearman correlation of 0.93, outperforming generalist models including LucaOne (0.90), ESM2-3B (0.84), and ESMC-600 M (0.71) (Fig. 5C). Notably, LucaVirus retained strong performance using nucleotide sequences as input (Spearman = 0.87), surpassing genome language models like Evo2-7B [13], DNABert2 [43] and Nucleotide Transformer-2.5B-multi-species [30] (Fig. 5C; Table S11), and demonstrating robust cross-modal consistency. These models were trained on early SARS-CoV-2 variants and tested on later, unseen variants, highlighting LucaVirus’s ability to extrapolate from limited experimental data to emerging strains.

To assess generality beyond coronaviruses, we extended the evaluation to additional viral families, including Enterovirus polyprotein fitness (Fig. 5D) and the influenza A hemagglutinin (HA) immune escape landscapes (Fig. 5E). LucaVirus consistently achieved higher correlation across both tasks compared to the tested baselines, confirming its broad applicability across viral families.

Finally, we evaluated LucaVirus’s intrinsic fitness awareness using the zero-shot ProteinGYM benchmark [44]. LucaVirus demonstrated competitive performance with state-of-the-art models—including the multiple sequence alignment (MSA)-dependent EVE [45] and Tranception series models [46], and the 98B-parameter ESM3 [12]—despite operating without alignment constraints and with significantly fewer parameters. This result reflects expected architectural trade-offs: EVE explicitly models evolutionary history via MSAs, while ESM3 leverages a massive parameter space (98B vs. 1B for LucaVirus); however, these computationally intensive strategies still conferred no statistically significant advantage over LucaVirus (*t*-test, *P* > 0.05).

### Challenge 4: Accurate antibody–antigen interactions modeling for pandemic readiness

**Figure 6.**
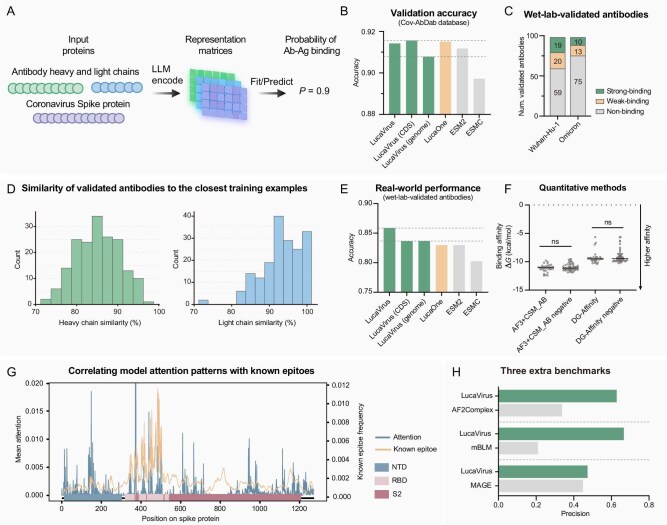
Accurate prediction of virus-specific antibody–antigen interactions. (A) Schematic diagram of the antibody–antigen binding prediction task. (B) Validation accuracy on the CoV-AbDab database. (C) Experimental validation using real-world antibody test data, illustrating the distribution of antibodies binding to the S protein of Wuhan-Hu-1 and Omicron strains in this data set. (D) Histograms showing the sequence similarity of validated antibodies to the closest examples in the training set. (E) Real-world performance accuracy on the wet-lab validated antibody set. (F) Predictions of two qualitative approaches on real-world data sets. (G) Attention analysis of the model. The line plot overlays the mean attention weights of the model onto the SARS-CoV-2 spike protein sequence. High attention regions (blue) correlate strongly with known epitopes (orange) in the N-terminal domain and receptor binding domain. (H) Benchmarking against three independent, state-of-the-art models. Precision (true positive/all predicted positive) is used to indicate the model’s efficacy in identifying novel, true-positive binders.

LucaVirus also demonstrated strong capability in identifying antibodies from repertoires targeting emerging viruses (Fig. 6A). Evaluated on the CoV-AbDab coronavirus antibody database [47], LucaVirus achieved 93% accuracy in predicting binding between antibodies and the SARS-CoV-2 spike protein (Fig. 6B; Table S9), indicating robust baseline performance.

To assess real-world utility, we simulated an antibody discovery pipeline using human B-cell receptor repertoires. We randomly selected 98 antibodies from single B-cell sequencing of convalescent COVID-19 patients and experimentally measured their binding to both Wuhan-Hu-1 and Omicron spike proteins (Fig. 6C; Fig. S12; Tables S12 and S13; Extended Data Set 4). After stringent filtering to exclude antibody with fewer than 10 amino acid mismatches from training data, 80 validated antibodies were retained for evaluation (Fig. 6D). On this data set, LucaVirus achieved accuracies of 84.6% (Wuhan-Hu-1) and 89.8% (Omicron), outperforming the protein language models tested (Fig. 6E) and structure-based methods—including AlphaFold 3 [48] combined with CSM-Ab [49] and DG-affinity [50] (Fig. 6F). Attention-weight visualization further revealed strong concordance between high-attention regions and known spike epitopes, suggesting that LucaVirus explicitly captures biologically relevant interaction sites (Fig. 6G).

Notably, LucaVirus demonstrates cross-modal flexibility. When retrained to predict binding affinity using spike-coding CDS sequences or entire viral genomes instead of protein sequences, the model maintained high accuracy (both 84.3%; Fig. 6D; Table S9), demonstrating its ability to extract antigenic signals directly from raw genomic data and streamline discovery pipelines where protein annotation may be missing.

Finally, we benchmarked LucaVirus against three state-of-the-art models—mBLM [51], AF2Complex [52], and MAGE [53]—across three independent data sets (Fig. 6H). LucaVirus consistently outperformed these competitors, achieving precisions ranging from 47% to 66%. In practical terms, this translates to an experimental hit rate of approximately one in every two candidates, providing a promising computational approach to prioritize candidates for therapeutic antibody screening. Crucially, all benchmark sequences were stringently filtered to ensure at least 10 mismatches from the training set, underscoring the robustness and generalization capability of LucaVirus in antibody-antigen interaction prediction.

## DISCUSSION

Modeling the evolutionary and functional landscape of viruses remains a fundamental challenge, largely due to their extensive sequence diversity and the limited biological understanding compared to cellular organisms. To address this gap, we present LucaVirus—a unified, multi-modal foundation model purpose-built for virology. By integrating nearly all available viral sequence data across both nucleotide and protein modalities, LucaVirus establishes a comprehensive framework for interpreting viral biology at multiple levels, from virus discovery and functional annotation to fitness landscape modeling and antibody–antigen interaction prediction.

Our results highlight the advantages of domain-specific foundation models as a valuable complement to general-purpose LLMs. While generalist models offer broad biological coverage, their substantial computational costs and lack of domain granularity often constrain their practical deployment in specialized fields [27,54]. In our comprehensive benchmarking, the 1B-parameter LucaVirus consistently matched or outperformed a range of leading generalist models—including both protein-centric (e.g. the ESM series) and genome-centric (e.g. Evo, Nucleotide Transformer) architectures—across diverse virological tasks. This underscores that domain specialization can offer measurable improvements in both performance and parameter efficiency.

Beyond benchmark performance, LucaVirus provides a window into the biological organization of viral sequence and function through its learned representations. The strong correlation between embedding geometry and genetic distance indicates that the model’s internal representation space recapitulates known evolutionary relationships. Critically, this correlation holds on remote sequences entirely absent from training, suggesting that LucaVirus captures intrinsic evolutionary forces, rather than data-set-specific artifacts. Furthermore, the model resolves functional genomic architecture at single-codon resolution, distinguishing coding from non-coding regions, identifying codon phase structure, and differentiating synonymous codons. This granularity demonstrates that multi-modal pre-training distills biologically meaningful structural principles into the model’s latent space.

Our ablation analyses provide insight into the architectural requirements for viral modeling. Both the unified nucleotide-protein representation and the semi-supervised, biologically informed pre-training objective proved essential for strong performance in supervised downstream tasks, consistent with prior studies [20,27,28]. Interestingly, in zero-shot fitness prediction, the pure mask-prediction variant slightly outperformed the standard LucaVirus, reflecting fundamental trade-off. While mask-prediction objective optimizes perplexity and benefits zero-shot probability estimation, the semi-supervised strategy explicitly forces the model to learn sequence–function associations, yielding superior performance in biologically relevant supervised tasks. This distinction underscores a key difference between biological sequences and natural language. Unlike natural language, which is largely self-descriptive [55], biological sequences encode function implicitly and context-dependently. Pure autoregression or masking may capture statistical patterns but fail to recover functional semantics. By incorporating explicit functional supervision, LucaVirus extracts biologically meaningful representations—capturing codon structure, synonymous mutations, and phylogenetic relationships—that form a stronger foundation for addressing complex, label-dependent virological challenges.

We further validate the effectiveness of transfer learning in biological sequence modeling. A central concern in fine-tuning is ‘catastrophic forgetting’ [29], whereby specialization erodes general knowledge. By benchmarking LucaVirus on the original LucaOne’s ten general biological tasks [20], we show LucaVirus retains core biological syntax while gaining virus-specific expertise. This establishes a robust and generalizable paradigm: initializing from a broad biological foundation and subsequently refining with domain-specific pre-training offers an effective strategy for building specialized biological AI models. The architectural decision to employ a bidirectional encoder (BERT-style) rather than a unidirectional generative decoder (GPT-style) was strategic. While generative architectures (GPT-like) have demonstrated utility in de novo sequence design by optimizing likelihood estimation [46,56], they rely on unidirectional causal masking which limits access to global contextual information. In contrast, our encoder-only architecture leverages bidirectional attention to capture the epistatic interactions and long-range dependencies essential for functional characterization [11]. Crucially, this architecture serves a ‘safety-by-design’ principle. By restricting the model to representation learning and analytical inference, we deliberately exclude the generative capabilities required for *de novo* pathogen design [57].

The capabilities demonstrated by LucaVirus have direct implications for both basic virology and pandemic preparedness. Fitness landscape prediction enables prospective mapping of mutational escape trajectories before antigenic variants emerge in surveillance data, providing actionable intelligence for vaccine and therapeutic design. The antibody–antigen prediction module achieves an experimental hit rate of approximately one in two candidates and maintains high accuracy when operating on raw genomic sequences rather than protein sequences, streamlining discovery pipelines where protein annotation may be incomplete. Beyond these specific applications, LucaVirus serves as a computational framework for generating and prioritizing biological hypotheses at scale, enabling rapid in silico exploration of mutational effects and candidate antibody–antigen pairs before resource-intensive experimental validation. We envision a surveillance-to-response pipeline in which LucaVirus continuously monitors metagenomic streams for novel viral emergence, predicts functional consequences of detected variants, and prioritizes candidates for experimental characterization—representing a foundational step toward scalable, AI-driven pandemic intelligence.

Several limitations warrant discussion. As a predictive model of viral fitness and host adaptation, LucaVirus may carry dual-use potential; we have implemented multiple safeguards including an encoder-only architecture that fundamentally excludes de novo sequence generation, structured access controls, and data desensitization protocols that provide predictions at the protein family level rather than as pathogen-specific mutation lists. The scope of experimental validation remains limited—while we validated candidate proteases experimentally, the majority of computational predictions require further verification, particularly for sequences in the homology twilight zone. Performance is bounded by the OpenVirus training corpus, and viral families from under sampled environments may yield lower-confidence predictions, necessitating periodic retraining as the known virosphere expands. For fitness landscape prediction, accuracy may decrease in regions of high epistatic complexity with sparse training data; for antibody–antigen prediction, performance may be limited for underrepresented antibody classes and does not account for post-translational modifications or glycan shielding. Addressing these limitations through expanded training data, architectural modifications, and integration with experimental feedback loops represents a clear priority. Looking forward, future integration of structural, host, and ecological information may further enhance LucaVirus’s ability to anticipate viral emergence and inform public health preparedness.

## MATERIALS AND METHODS

Detailed materials and methods are available in the Supplementary Data.

## Supplementary Material

nwag376_Supplemental_File

## Data Availability

All data, code, and materials used in this analysis are publicly available to researchers for reproducing or extending the findings. Pretrained weights for the LucaVirus foundation model, downstream models, and the training data set are accessible on Zenodo (DOI: 10.5281/zenodo.15703216). Raw sequencing reads from the independent wetland sediment NGS data set have been deposited in the CNGBdb under project accession number CNP0009505.

## CODE AVAILABILITY

The source code for modeling and pretraining, along with instructions for obtaining pretrained weights, is accessible at https://github.com/LucaOne/LucaVirus. The code for downstream task modeling and instructions for retrieving trained model weights are available at https://github.com/LucaOne/LucaVirusTasks.
