# Supplementary material for: Predicting the evolutionary and functional landscapes of viruses with a unified nucleotide-protein language model: LucaVirus
Source: Natl Sci Rev. 2026 Jun 17;13(14):nwag376. doi: 10.1093/nsr/nwag376 (PMC13397533; doi:10.1093/nsr/nwag376)
Supplement: nwag376_Supplemental_File [file nwag376_supplemental_file.docx]

Supplementary Materials for

**Predicting the Evolutionary and Functional Landscape of Viruses with a Unified Genome-Protein Language Model: LucaVirus**

Yuan-Fei Pan^1,2,3^†*, Yong He^4^†*, Yu-Qi Liu^5,6,7,8^, Yong-Tao Shan^8,9^, Shu-Ning Liu^10^, Jia-Hao Ma^2,3^, Xue Liu^2^, Xiaoyun Pan^1^, Yinqi Bai^11^, Zan Xu^4^, Tingjun Hou^12^, Zheng Wang^4^, Jieping Ye^4^, Jianguo He^9,13^, Edward C. Holmes^14^, Bo Li^1,15^*, Yao-Qing Chen^10^*, Zhao-Rong Li^4,8^*, Mang Shi^2,3,16^*‡

*Corresponding authors: yfpan21@m.fudan.edu.cn (Y.-F.P.); sanyuan.hy@alibaba-inc.com (Y.H.); bool@fudan.edu.cn (B.L.); chenyaoqing@mail.sysu.edu.cn (Y.-Q.C.); lzr098@gmail.com (Z.-R.L.); shim23@mail.sysu.edu.cn (M.S.).

†Equally contributed to this work.

‡Lead contact.

**The PDF file includes:**

**I. Methods**

**II. Supplementary Figures:** Figs. S1 to S11

**III. Supplementary Tables:** Tables S1 to S13

**I. Methods**

**Data Collection and Pre-processing**

**The OpenVirus Corpus.** We curated OpenVirus, a comprehensive data set of viral sequences used to train the LucaVirus model. This data set comprises 15.7 million viral sequences, totaling 25.4 billion tokens—including 23.7 billion nucleotide tokens from 10.4 million sequences and 1.6 billion amino acid tokens from 5.2 million protein sequences. Nucleotide sequences were collected from NCBI Virus and seven large-scale virus discovery studies [1-6] to ensure coverage of uncatalogued viral diversity. Protein sequences were obtained from UniProtKB [7] and the ColabFold environmental database (envdb) [8], which integrate and de-redundifies large environmental databases such as BFD and MGnify [9]. To mitigate sampling bias and capture intra-species variation, nucleotide sequences were clustered using MMseqs2 [10] easy-cluster at 80% coverage and 80% ANI. Up to 10 non-redundant sequences were sampled per cluster, ensuring balanced representation across species-level taxa defined by 80% ANI. The final data set spans all known viral realms (including *Heunggongvirae*, *Orthornavirae*, and *Pararnavirae*) and encompasses virus-infecting hosts across all cellular domains.

**Biological Annotations.** To support biologically informed pre-training, we enriched the sequence data with functional and taxonomic annotations. For nucleotide sequences, annotations were primarily derived from GenBank GBFF files, from which coding region coordinates and order-level taxonomic labels were extracted. Sequences lacking annotations were retained with missing fields left blank. Protein annotations were obtained from UniProtKB [7] and InterPro [11], with InterPro providing details on functional sites, conserved domains, and homologous superfamilies. Taxonomic labels were defined at the order level based on 63 categories, with missing entries assigned a default label of -100. Functional annotations included: (i) 946 functional site categories (e.g. active sites, binding sites, conserved sites, and post-translational modifications), (ii) 3,460 homologous superfamilies, (iii) 1,659 conserved domains, and (iv) 603 functional keyword groups.

**Model Architecture and Pre-training**

**Architecture.** LucaVirus is a 1-billion-parameter, encoder-only Transformer model purpose-built for viral sequence analysis. It features 12 transformer layers, a hidden dimension of 2,560, and an expanded context window of 3,072 tokens. We strategically adopted an encoder-only architecture rather than a generative framework for two reasons: (i) Interpretability: encoder models extract robust feature matrices rather than generating text, enabling direct interpretation and analysis of learned biological features; and (ii) Biosecurity: by design, encoder-only models lack generative capacity, thereby mitigating dual-use risks associated with *de novo* pathogen sequence generation.

**Model Scaling and Structural Adaptations.** We determined the model’s parameter size by adopting a parameter-to-token ratio of approximately 1:25 relative to our available training data. This yielded a 1B-parameter architecture well supported by the OpenVirus corpus, which contains twice the non-redundant tokens of NCBI Virus and 30% more than UniProtKB. To reduce parameter relative to the 1.8B LucaOne parent model [12] while preserving representational capacity, we retained the high-dimensional embedding size (2,560) but reduced the network depth (from 20 to 12 layers) and the prediction head size (from 40 to 20). To better capture long-range dependencies characteristics of viral genomes, we extended the maximum context length from 1,280 to 3,072 tokens. This expansion, optimized for NVIDIA A100 (80GB) hardware, accommodates the full-length sequences of 99.1% of full-length viral proteins in SwissProt [7].

**Transfer Learning and Pre-training Protocol.** LucaVirus was initialized from LucaOne checkpoint at step 1,760,000 [12] and subsequently refined using a semi-supervised pre-training strategy. This checkpoint represented the latest version of the general foundation model at the time of project initiation. This approach enables inheritance of broad biological sequence priors followed by virus-specific specialization. The pre-training process integrated self-supervised masked language modeling (MLM) with seven biologically relevant supervised tasks, including sequence-level classification (order-level taxonomy for nucleotides/protein sequences and functional keyword prediction) and token-level annotation (gene boundaries, homologous superfamilies, conserved domains, and functional sites). To balance these objectives, we assigned a loss weight of 1.0 for MLM and keyword prediction tasks, and 0.2 for the remaining auxiliary tasks.

Training was conducted on eight NVIDIA A100 GPUs (Alibaba Cloud) for 70 days, using a learning rate of 2×10^-4^ with warm-up phase, a batch size of 8, and gradient accumulation 32 steps. The model was trained for 3.8 million steps (approximately 1.9 epochs), processing over 49 billion tokens. Convergence was monitored by tracking average loss every 4,000 steps.

Benchmarking across all downstream tasks confirmed that both the unified nucleotide–protein representation and the semi-supervised training objectives are essential for optimal performance. In addition, evaluation on general biological benchmarks from the original LucaOne study verified that the transfer learning strategy effectively avoids catastrophic forgetting.

**Supervised label provenance and data leakage prevention.** The semi-supervised pre-training strategy incorporates biologically supervised objectives alongside masked language modeling. The supervised labels and their sources are as follows: (i) order-level taxonomic labels were obtained from NCBI GenBank and UniProtKB release 2024_06 annotations; (ii) functional keyword annotations were derived from UniProtKB keyword categories (UniProtKB controlled vocabulary); (iii) protein domain and active-site annotations were sourced from InterPro release 96.0. Ideally, all of these label sources are independent of the downstream evaluation datasets used in this study. However, we acknowledge that some supervised labels may co-vary with downstream task labels; for example, capsid and enzyme function predictions may correlate with protein domain annotations or taxonomic annotations. Accordingly, in addition to standard train–test splits, we applied further filtering to exclude test samples with significant hits to the pre-training dataset. Details are described in the corresponding section for each downstream task.

**Ablation Studies.** To validate our architectural and training strategies, we developed three ablation variants: LucaVirus-Prot (protein-only), LucaVirus-Nucl (nucleotide-only), and LucaVirus-Mask (masked language modeling only, without additional biological supervision). We benchmarked these variants against the standard LucaVirus model across all downstream tasks (detailed below).

To assess potential “catastrophic forgetting”—the erosion of fundamental biological capabilities during domain-specific pre-training—we compared LucaVirus against the original LucaOne foundation model [12]. We employed the standardized evaluation suite established in the LucaOne study, which encompasses ten general biological tasks across three domains: (1) Fundamental Sequence-Function Mapping: We utilized the Central Dogma task to assess the model's intrinsic ability to map DNA sequences to protein products without explicit alignment supervision. Additionally, we evaluated Gene Taxonomy Prediction (at Superkingdom, Species, and Genus levels) to verify the retention of phylogenetic signals. (2) Single-Molecule Property Prediction: To confirm the preservation of molecular feature recognition, we included Non-coding RNA Family classification (ncRNAFam) for RNA structural semantics, Prokaryotic Protein Subcellular Localization (ProtLoc) for sorting signal identification, and Protein Stability (ProtStab) regression for biophysical property estimation. (3) Molecular Interaction Prediction: To evaluate cross-modal and pairwise interaction capabilities, we tested Protein-Protein Interaction (PPI), ncRNA-Protein Interaction (ncRPI), and Influenza A Antigenic Relationship (InfA) prediction.

**Embedding Analyses**

**Nucleotide Representation and Biological Syntax.** To visualize the nucleotide-level representations learned by LucaVirus, we extracted embedding matrices from 30 annotated Enterovirus reference genomes obtained from NCBI/GenBank. Dimensionality reduction was performed using Principal Component Analysis (PCA). To assess biological interpretability, embeddings were colored by four attributes: (i) nucleotide identity (A, T, C, G), (ii) codon phase (1st, 2nd, or 3rd position), (iii) encoded amino acid, and (iv) synonymous codon identity.

To quantitatively validate these observations, we analyzed an independent data set of 3,765 viral sequences from held-out development and test sets. We employed permutational multivariate analysis of variance (PERMANOVA) [13] to test for significant differences in embedding clusters corresponding to nucleotide identity, codon position, and coding semantics. Furthermore, we conducted linear probing experiments to assess the linear separability of these biological features, in which a logistic regression classifier was trained on the frozen embeddings. We benchmarked LucaVirus against state-of-the-art genomic models, including Nucleotide Transformer [14], Evo [15], Evo2 [16], and LucaOne [12].

Finally, we evaluated whether LucaVirus captures virus-enriched genomic features associated with programmed ribosomal frameshifting (PRF). Specifically, 300-bp windows centered on annotated PRF sites were extracted from the FSDB database [17]. We evaluated whether embedding’s ability to distinguish the relative position of nucleotides within a codon (codon phase). A correctly learned representation should reflect the phase shift inherent in the biological mechanism; failure to identify the frameshift would result in misaligned phase predictions. We compared the codon phase discrimination performance of LucaVirus against baseline models using the linear probing method described above.

**Protein Representation and Family Discrimination.** To evaluate protein-level representation capacity, we compared LucaVirus against mainstream models trained across all domains of life (including eukaryotic viruses, which are underrepresented or partially excluded in some models), namely, LucaOne [12], ESM2 [18], and ESMC [19]. Viral reference protein sequences were retrieved from NCBI RefSeq and grouped into families using the VOGDB pipeline [20], which employs DIAMOND [21] for pairwise alignment followed by Markov clustering [22]. We selected clusters containing >20 sequences and randomly sampled 10 families, from which 10 sequences were randomly sampled (N=100 total), and per-residue feature embeddings were extracted. PCA was then applied to visualize separation of protein families within the learned representation space.

**Correlation with Evolutionary Divergence.** To quantify the relationship between LucaVirus embeddings and evolutionary divergence, we performed correlation analyses between embedding space distances (cosine and Euclidean) and genetic divergence metrics (p-distance and maximum likelihood estimates of substitution rates). The primary data set comprised 5,566 viral orthologous protein families with corresponding coding DNA sequences (CDS) from RefSeq. To evaluate generalization to previously “unseen” viral diversity, we additionally included 938 protein families from novel RNA viruses (“dark matter” viruses) identified in our prior study [1], which exhibit no significant BLAST hits to the pre-training corpus. Protein families were defined using *de novo* clustering via the VOGDB pipeline [20]. For each family, mean pairwise embedding distances were computed and correlated with genetic distances derived from multiple sequence alignments (MSA).

**LMAlign: Embedding-Based Sequence Alignment and Validation.** To assess whether LucaVirus representations capture deep evolutionary signals, we developed LMAlign, an embedding-based pairwise sequence alignment algorithm. Input sequences are first processed by LucaVirus to generate dense embedding matrices of size $L\times2560$. A dynamic similarity matrix ($L_{1}\times L_{2})$ is then computed using cosine similarity between residue-level embeddings, which serves as the scoring matrix for dynamic programming. This framework enables optimal local and global alignments analogous to the Smith-Waterman and Needleman-Wunsch algorithm respectively, but directly from the model’s semantic feature space.

LMAlign was benchmarked for remote homology detection using the SCOPe 2.08 data set [23] filtered for viral proteins. To evaluate performance across varying degrees of evolutionary distance, test pairs were stratified across three hierarchical levels: family, superfamily, and fold. We performed all-against-all pairwise alignments, comparing LMAlign (local and global modes) with conventional Needleman-Wunsch and Smith-Waterman alignments utilizing BLOSUM62 [24] and BLASTP [25] scores. Performance was evaluated using receiver operating characteristic (ROC) curves and errors per query (EPQ) metrics, quantifying sensitivity in detecting homologous relationships beyond the reach of sequence-identity-based methods.

**A General Modeling Framework for Downstream Tasks**

**Unified Model Architecture.** We implemented a standardized framework to adapt pre-trained models for diverse downstream applications. The core architecture consists of a frozen pre-trained encoder (e.g., LucaVirus) for embedding extraction, followed by a trainable value-level attention pooling (VALP) layer [26] and a fully connected (FC) prediction head. This design concentrates task-specific learning in a lightweight head while preserving the generality and stability of the pre-trained backbone. The framework supports two input modes: (1) Single-Sequence Mode: Processes individual sequences (e.g., for function prediction). It combines the frozen embedding pathway with an optional, trainable lightweight Transformer encoder to capture task-specific features. (2) Multi-Sequence Mode: Processes interacting sequences (e.g., for antibody-antigen binding). Each sequence is encoded and pooled independently; the resulting vectors are concatenated to form a joint representation. The final FC head is flexible and supports binary, multi-class, or multi-label classification, as well as regression by adjusting output dimensions.

**Training and Reporting Protocol.** To ensure rigorous and unbiased evaluation, all data sets were strictly partitioned into independent training, development, and test sets. The model was trained using the AdamW optimizer. For each task, we conducted a grid search over learning rates (1×10^-4^, 2×10^-4^) and batch sizes (8, 16) to determine optimal training hyperparameters. Model selection was based on optimal performance on the development set, and final results are reported on the independent test set.

**Challenge 1: Identification of Viral “Dark Matter”**

**Data Set and Task Setup.** We formulated two binary classification tasks targeting viral hallmark proteins: RNA-dependent RNA polymerase (RdRp) and Capsids. The data set comprises 5,979 RdRp positives and 229,434 negatives sourced from previous studies, alongside 216,742 Capsid positives (UniProt KW2013) balanced against 83,461 hard negatives (e-value < 10^-5^ via DIAMOND [21] alignment against UniProtKB [7] and NCBI nr databases) and 133,281 easy negatives randomly sampled from non-capsid viral proteins on SwissProt. The data is randomly split into 75% training and 10% development, 15% testing sets. We employed the general modeling framework for downstream tasks described above configured with a binary classification head for these tasks.

**Validation on Viral “Dark Matter”.** To assess the model's capacity to detect remote viral homologs effectively (“dark matter”), we screened sequences from the LucaProt data set [1]. We specifically targeted sequences that lacked any significant hits (search method: mmseqs easy-search, sensitive level 7, e-value < 1e-5) to the capsid training dataset. To prevent potential data leakage and ensure a rigorous assessment of generalization, any sequences sharing ≥50% identity with the LucaVirus pre-training corpus were strictly excluded.

Candidates predicted as positive were clustered using DIAMOND BLASTP [21] and MCL [22]. Representative sequences from each cluster were subjected to structure prediction using ESMFold [18] and ColabFold [8], followed by FoldSeek [27] searches against PDB [28], UniRef50, and SwissProt [7] (e-value < 1e-5) to verify structural homology to known capsids. High-confidence candidates were further validated via high-accuracy structure prediction using AlphaFold3 [29].

While numerous proteins in the dark matter exhibited no significant structural hits or matched structures of unknown function, we identified a high-confidence set of 115 structurally validated capsid proteins. This set serves as the primary benchmark for comparing LucaVirus against other AI-driven and traditional bioinformatics approaches

**Benchmarking against Bioinformatic Approaches.** To rigorously evaluate LucaVirus against standard bioinformatics pipelines, we conducted a comparative benchmark using sequence similarity and structure-based search tools. We compared LucaVirus against DIAMOND BLASTP (ultra-sensitive mode) [21], MMseqs2 profile search (sensitivity -s 7) [10], HHblits (10 iterations) [30], and FoldSeek (using ProstT5 module [31] for structure prediction) [27], other parameters not listed in the panthers are set to default values of the corresponding software. To ensure a fair comparison, the database for all retrieval methods was constructed exclusively from the LucaVirus training set. For the above methods, we defined two classification criteria using an e-value threshold 10^-5^: (1) Top-hit Mode: Classification is determined solely by the hit with the highest bitscore. If the top hit is a capsid, the query is classified as positive. (2) Strict Consensus Mode: A query is classified as positive only if all significant hits (e-value < 10^-5^) are annotated as capsids; otherwise, it is classified as negative.

**External validation on independent coastal metatranscriptomes.** To rigorously evaluate the real-world generalizability of LucaVirus, we leveraged an independent, newly derived metatranscriptomic dataset sequencing from wetland sediments (comprising 20 samples with 273.9 Gbp of raw reads). This dataset represents a complex, pristine ecosystem entirely absent from the OpenVirus corpus used for model training.

Raw sequencing reads underwent stringent quality control, adapter trimming, and de novo metatranscriptomic assembly following our previously established pipeline [32]. Open reading frames (ORFs) across all assembled contigs were predicted using ORFfinder with default parameters to capture all potential protein-coding sequences. To optimize computational efficiency and reduce inference time for the downstream deep-learning pipeline, the predicted ORFs were clustered using MMseqs2 linclust with a stringent threshold of 50% sequence identity and 80% bidirectional alignment coverage. The resulting cluster representatives were then subjected to the LucaVirus hallmark protein identification model to predict putative viral capsid proteins.

**Multi-Tier Independent Validation of Putative Viral Capsids.** We implemented a rigorous evidence classification system based on structural, sequence, and genomic contexts: Structural Confirmation (Tier 1): For unclassified candidates showing low sequence similarity to known proteins, full-length 3D structural models were predicted in silico using ESMFold. The generated structures were then used as queries in FoldSeek to search against comprehensive structural databases, including the Protein Data Bank (PDB), SwissProt, and the Big Biological Macromolecule FoLd space database (BFVD). A structural match with a structural similarity score surpassing known viral capsid folds was considered direct, homology-independent proof of a viral origin. Virus-associated Sequence and Domain Evidence (Tier 2): Sequence-level validation was performed by combining taxonomic and functional annotations. The predicted sequences were searched against the NCBI Non-Redundant (NR) protein database using DIAMOND blastp to detect remote viral homologues. Simultaneously, functional domains were profiled using InterProScan to identify capsid-specific signatures or viral motifs. Furthermore, genomic context was evaluated by examining the parent contigs; sequences were validated if they were co-located with well-established virus-specific hallmark genes (e.g., RNA-dependent RNA polymerase [RdRp], terminase, or portal proteins) on the same contig.

For benchmarking comparisons, the subset of sequences verified by Tier 1 and Tier 2 criteria was extracted to serve as an empirical, stringently validated "gold standard" dataset to evaluate the accuracy, sensitivity, and baseline alignments of LucaVirus against alternative artificial intelligence models and traditional bioinformatics toolsets.

**Computational Efficiency Analysis.** To evaluate scalability and runtime performance, we recorded the mean per-sequence query time and total database preparation/training time. To ensure a fair comparison between AI-driven and traditional algorithms, we utilized cost-equivalent hardware configurations: the AI models were tested on a single NVIDIA A100 (80GB) GPU, while CPU-based bioinformatics tools utilized dual-socket AMD EPYC 7H12 CPUs (128 cores total), with all tools configured to use 128 threads to fully saturate the CPU resources.

**Challenge 2: Characterization of Unknown Proteins**

**Data Set and Task Setup.** We formulated a multi-label classification task utilizing 10,715 viral proteins from UniProtKB [7] annotated with Enzyme Commission (EC) numbers, filtering for labels with at least 100 associated sequences to ensure enough training samples per label. 70% training and 15% development, 15% testing sets. The model employed the general modeling framework for downstream tasks, configured with a sigmoid activation multi-label prediction head to output probabilities for enzyme classes.

**Benchmarking on Structure-Inferred Annotations.** To evaluate the model’s capacity to annotate proteins that lack sequence homology to known enzymes, we utilized the structurally defined viral data set from ref [33]. In this data set, ground truth labels were generated by propagating InterProScan [34] functional annotations (CDD [35], TIGRFAM [36], Pfam [37]) within structural clusters defined by ColabFold [8] and FoldSeek [27] (propagation threshold: >25% intra-cluster consistency). To ensure a rigorous test of generalization, we strictly excluded all test sequences sharing >50% sequence identity with the LucaVirus pre-training corpus. We compared LucaVirus against ESM2-3B [18] and the original structure-based propagation method, reporting standard classification metrics (accuracy, precision, recall, F1) as well as annotation coverage—defined as the proportion of total proteins successfully assigned a specific EC label.

**Experimental Validation of Putative Proteases.** To validate the model’s predictions, RNA viral proteins predicted to be proteases (EC 3.4.-.-) from the LucaProt study, which identified distant homologs of dark matter RNA virus proteins, were selected. Candidates were clustered into families (following the VOGDB methodology [20]), and two representatives from distinct families: CL6_PICL_PRO (22.4 kDa) and CL31_SOBL_PRO (21.7 kDa), were selected for characterization.

**Protein Expression and Purification.** Genes were cloned into expression vectors and transformed into *Escherichia coli* BL21 (DE3). Cultures were grown in LB medium at 37°C until OD_600_ ≈ 0.6, induced with 0.5 mM IPTG, and incubated at 16°C for 16 hours. Cells were harvested and resuspended in Lysis Buffer (20 mM Tris-HCl, 400 mM NaCl, 20 mM imidazole, pH 7.5). Following high-pressure homogenization (700 Pa) and clarification by centrifugation (16,000 rpm, 25 min, 4°C), the supernatant was loaded onto nickel affinity resin. After washing with 10 column volumes of Lysis Buffer, the 12His-SUMO tag was cleaved on-column by overnight protease digestion in Elution Buffer (50 mM Tris-HCl, 100 mM NaCl, pH 7.0) to elute the native protein.

**Functional Assays.** Protein purity was verified via SDS-PAGE on 4–20% pre-cast gels. Proteolytic activity was quantified using a fluorescence-based protease assay (Beyotime, Catalog No. P0403S) according to the manufacturer’s protocol. Proteinase K was included as a positive control to verify assay sensitivity and to provide a reference activity curve. Purified target proteases expressed from empty vector backbone (lacking the protease insert) were included as negative controls to account for background signal from the expression system. Inactivated mutants (active-site serine-to-alanine substitutions) for two of the six candidate proteases were additionally included to confirm that observed activity was attributable to catalytic function rather than nonspecific effects. Cross-comparison of the resulting activity curves (Fig. 4G) confirmed that all six candidate proteases exhibited dose-dependent activity profiles comparable to the Proteinase K positive control, while both empty-vector and inactive-mutant controls showed negligible signal above background.

**Challenge 3: Forecasting Virus Evolvability**

**Supervised Fitness Prediction.** We formulated the prediction of viral fitness landscapes as a scalar regression problem utilizing the general modeling framework for downstream tasks. To rigorously evaluate the model’s capacity to forecast evolutionary trajectories across distinct viral families and selection pressures, we curated three comprehensive Deep Mutational Scanning (DMS) data sets:

**SARS-CoV-2 Receptor Binding (Temporal Generalization).** We utilized DMS data measuring the ACE2 binding affinity of the SARS-CoV-2 receptor-binding domain (RBD) [38]. To simulate a realistic forecasting scenario, we adopted a temporal split strategy: the model was trained on early variants (Wuhan-Hu-1, Alpha, Beta; N=34,055) and evaluated on later-emerging variants (Delta, Iota; N=19,274). This design tests the ability to generalize fitness predictions to future evolutionary lineages.

**Influenza H3N2 Immune Escape (Antigenic Generalization).** To assess predictions of antigenic evolution, we incorporated the immune escape landscape of Influenza A virus (H3N2, strain A/Hong Kong/19/2014) [39]. The fitness metric is defined as the **e**scape fraction, calculated as the ratio of mutant abundance in serum-selected cultures versus non-selected controls. We implemented a cross-serum split: the model was trained on escape profiles derived from specific sera and tested on differing sera to evaluate its ability to capture transferable antigenic features independent of specific antibody repertoires.

**Enterovirus A Polyprotein (Indel Robustness).** To extend prediction capabilities beyond substitutions, we utilized a Deep Indel Scanning data set for the Enterovirus A polyprotein [40]. This data set measures fitness via relative abundance in cell culture following the introduction of single amino acid substitutions, insertions, and deletions. Given the high dimensionality and lack of replicates for specific mutants, we employed a randomized split (75% training, 10% development, 25% testing) to assess the model's robustness to structural perturbations including indels.

**Zero-Shot Fitness Estimation.** To benchmark the model’s intrinsic, unsupervised understanding of viral fitness, we performed zero-shot predictions on the viral subset of the ProteinGym benchmark [41], comprising 31 diverse viral protein assays. Consistent with established methodologies, fitness scores were computed as the masked token Log-Likelihood Ratio (LLR) between the mutant and wild-type sequences:

$$LLR_{i}^{wt\to mut}=\log P\left( x_{i}^{mut} | C \right)-\log P(x_{i}^{wt}|C)$$

Where $i$ denote the $i$^th^ amino acid within protein sequence, $wt$ denote wildtype residue, $mut$ denote mutant residue, $P\left( x | C \right)$ represents the model’s predicted probability of masked token $x$ given the unmasked context $C$.

**Challenge 4: Prediction of Antigen-Antibody Binding Affinity**

**Data Set and Task Setup.** We constructed a binary classification data set using positive samples from CoVAbDab [42] (human-derived, strong SARS-CoV-2 binders) and negative samples augmented from pre-pandemic human BCR sequences (assumed non-binders, 10x positive count). The data was split into 70% training and 30% testing sets. We employed the general modeling framework described above, concatenating pooled embeddings from antibody heavy chain, light chain, and antigen sequences to predict binding probability.

**Real-World Experimental Validation.** We established a validation pipeline from single-cell sequencing to functional ELISA. Single-cell V(D)J data from convalescent/vaccinated donors was processed (Cell Ranger, IgBLASTn [43]) to identify paired heavy/light chains. To prevent data leakage, we strictly excluded candidates with <10 amino acid mismatches relative to the training set. Selected candidates were synthesized, expressed in HEK293T cells, and purified using Protein-A agarose beads. Binding affinity was assessed via ELISA assay. High-binding microtiter plates (Costar) were coated with 2 µg/mL of SARS-CoV-2 WT Spike, WT RBD, or Omicron Spike recombinant proteins overnight at 4°C. Following blocking (3% BSA), purified mAbs (10 µg/ml, 1:3 serial dilution) were incubated for 1 hour at 37°C. Bound antibodies were detected using HRP-conjugated goat anti-human IgG (1:2000). OD_405_ exceeding twice the PBS blank control was defined as positive binding. Binding strength (AUC) was visualized via heatmaps generated in GraphPad Prism 8.0.

We utilized this in-house data set to benchmark LucaVirus against generalist protein language models (e.g., ESM2 [18], ESMC [19]). Additionally, we evaluated two quantitative structure-based prediction approaches: (1) AlphaFold 3+CSM-AB, a composite pipeline combining AlphaFold 3 [29] for structural modeling and docking with the CSM-AB [44] scoring function; and (2) DG-Affinity [45]. For these methods, we compared the predicted binding energies (or affinity scores) for experimentally validated binders versus non-binders. Statistical significance of the discrimination capability was assessed using independent *t*-tests.

**Comparative Benchmarking against Specialized Models.** We further benchmarked LucaVirus against three distinct classes of state-of-the-art models: (1) mBLM (protein language model) [46]: Evaluated on our in-house experimental data set. We aggregated mBLM’s multi-class outputs into a binary binding prediction to facilitate direct comparison. (2) MAGE (generative language model) [47]: Evaluated on 20 *de novo* antibodies generated by MAGE, predicting their binding to Wuhan-Hu-1 RBD/Spike. To evaluate generalization, we confirmed that the sequence identity of these test antibodies to the MAGE training set was higher than to the LucaVirus training set, implying a stricter test of generalization for LucaVirus. (3) AF2Complex (structure-based) [48,49]: Evaluated on 971 antibodies (471 binders, 500 non-binders) from the AF2Complex study, filtered to exclude sequences with <10 mismatches relative to our training data. We computed optimal thresholds (Youden's index) for AF2Complex’s continuous interaction scores to enable binary classification metric comparison.

**References**

1. Hou X, He Y, Fang P, et al. Using artificial intelligence to document the hidden RNA virosphere. *Cell*. 2024;187(24):6929-6942.e16. doi:10.1016/j.cell.2024.09.027

2. Zayed AA, Wainaina JM, Dominguez-Huerta G, et al. Cryptic and abundant marine viruses at the evolutionary origins of Earth’s RNA virome. *Science*. 2022/04/08 2022;376(6589):156-162. doi:10.1126/science.abm5847

3. Neri U, Wolf YI, Roux S, et al. Expansion of the global RNA virome reveals diverse clades of bacteriophages. *Cell*. 2022/10/13/ 2022;185(21):4023-4037.e18. doi:<https://doi.org/10.1016/j.cell.2022.08.023>

4. Gregory AC, Zayed AA, Conceicao-Neto N, et al. Marine DNA Viral Macro- and Microdiversity from Pole to Pole. *Cell*. May 16 2019;177(5):1109-1123 e14. doi:10.1016/j.cell.2019.03.040

5. Wolf YI, Silas S, Wang Y, et al. Doubling of the known set of RNA viruses by metagenomic analysis of an aquatic virome. *Nature Microbiology*. 2020/10/01 2020;5(10):1262-1270. doi:10.1038/s41564-020-0755-4

6. Camarillo-Guerrero LF, Almeida A, Rangel-Pineros G, Finn RD, Lawley TD. Massive expansion of human gut bacteriophage diversity. *Cell*. 2021/02/18/ 2021;184(4):1098-1109.e9. doi:<https://doi.org/10.1016/j.cell.2021.01.029>

7. The UniProt Consortium. UniProt: the universal protein knowledgebase. *Nucleic Acids Research*. 2017;45(D1):D158-D169. doi:10.1093/nar/gkw1099

8. Mirdita M, Schütze K, Moriwaki Y, Heo L, Ovchinnikov S, Steinegger M. ColabFold: making protein folding accessible to all. *Nature Methods*. 2022/06/01 2022;19(6):679-682. doi:10.1038/s41592-022-01488-1

9. Richardson L, Allen B, Baldi G, et al. MGnify: the microbiome sequence data analysis resource in 2023. *Nucleic Acids Research*. 2023;51(D1):D753-D759. doi:10.1093/nar/gkac1080

10. Steinegger M, Söding J. MMseqs2 enables sensitive protein sequence searching for the analysis of massive data sets. *Nature biotechnology*. 2017;35(11):1026-1028.

11. Hunter S, Apweiler R, Attwood TK, et al. InterPro: the integrative protein signature database. *Nucleic acids research*. 2009;37(suppl_1):D211-D215.

12. He Y, Fang P, Shan Y, et al. Generalized biological foundation model with unified nucleic acid and protein language. *Nature Machine Intelligence*. 2025/06/18 2025;doi:10.1038/s42256-025-01044-4

13. Anderson MJ. Permutational multivariate analysis of variance (PERMANOVA). *Wiley statsref: statistics reference online*. 2014:1-15.

14. Dalla-Torre H, Gonzalez L, Mendoza-Revilla J, et al. Nucleotide Transformer: building and evaluating robust foundation models for human genomics. *Nature Methods*. 2025;22(2):287-297.

15. Nguyen E, Poli M, Durrant MG, et al. Sequence modeling and design from molecular to genome scale with Evo. *Science*. 2024;386(6723):eado9336. doi:doi:10.1126/science.ado9336

16. Brixi G, Durrant MG, Ku J, et al. Genome modeling and design across all domains of life with Evo 2. *bioRxiv*. 2025:2025.02.18.638918. doi:10.1101/2025.02.18.638918

17. Moon S, Byun Y, Han K. FSDB: a frameshift signal database. *Computational Biology and Chemistry*. 2007;31(4):298-302.

18. Lin Z, Akin H, Rao R, et al. Evolutionary-scale prediction of atomic-level protein structure with a language model. *Science*. 2023/03/17 2023;379(6637):1123-1130. doi:10.1126/science.ade2574

19. Hayes T, Rao R, Akin H, et al. Simulating 500 million years of evolution with a language model. *Science*. 2025;387(6736):850-858. doi:doi:10.1126/science.ads0018

20. Trgovec-Greif L, Hellinger H-J, Mainguy J, et al. VOGDB—Database of Virus Orthologous Groups. *Viruses*. 2024;16(8):1191.

21. Buchfink B, Reuter K, Drost H-G. Sensitive protein alignments at tree-of-life scale using DIAMOND. *Nature Methods*. 2021/04/01 2021;18(4):366-368. doi:10.1038/s41592-021-01101-x

22. Van Dongen S. Graph Clustering Via a Discrete Uncoupling Process. *SIAM Journal on Matrix Analysis and Applications*. 2008;30(1):121-141. doi:10.1137/040608635

23. Fox NK, Brenner SE, Chandonia J-M. SCOPe: Structural Classification of Proteins—extended, integrating SCOP and ASTRAL data and classification of new structures. *Nucleic Acids Research*. 2013;42(D1):D304-D309. doi:10.1093/nar/gkt1240

24. Henikoff S, Henikoff JG. Amino acid substitution matrices from protein blocks. *Proceedings of the National Academy of Sciences*. 1992;89(22):10915-10919.

25. Altschul SF, Gish W, Miller W, Myers EW, Lipman DJ. Basic local alignment search tool. *Journal of molecular biology*. 1990;215(3):403-410.

26. He Y, Wang C, Zhang S, Li N, Li Z, Zeng Z. KG-MTT-BERT: Knowledge graph enhanced BERT for multi-type medical text classification. *arXiv preprint*. 2022;arXiv:2210.03970

27. van Kempen M, Kim SS, Tumescheit C, et al. Fast and accurate protein structure search with Foldseek. *Nature Biotechnology*. 2024/02/01 2024;42(2):243-246. doi:10.1038/s41587-023-01773-0

28. Burley SK, Berman HM, Kleywegt GJ, Markley JL, Nakamura H, Velankar S. Protein Data Bank (PDB): the single global macromolecular structure archive. *Protein crystallography: methods and protocols*. 2017:627-641.

29. Abramson J, Adler J, Dunger J, et al. Accurate structure prediction of biomolecular interactions with AlphaFold 3. *Nature*. 2024/06/01 2024;630(8016):493-500. doi:10.1038/s41586-024-07487-w

30. Steinegger M, Meier M, Mirdita M, Vöhringer H, Haunsberger SJ, Söding J. HH-suite3 for fast remote homology detection and deep protein annotation. *BMC Bioinformatics*. 2019/09/14 2019;20(1):473. doi:10.1186/s12859-019-3019-7

31. Heinzinger M, Weissenow K, Sanchez J, Henkel A, Steinegger M, Rost B. Prostt5: Bilingual language model for protein sequence and structure. bioRxiv, 2023. *URL* [*https://www*](https://www) *biorxiv org/content/early/2023/07/25/202307*. 23

32. Pan Y-F, Zhao H, Gou Q-Y, et al. Metagenomic analysis of individual mosquito viromes reveals the geographical patterns and drivers of viral diversity. *Nature Ecology & Evolution*. 2024/05/01 2024;8(5):947-959. doi:10.1038/s41559-024-02365-0

33. Nomburg J, Doherty EE, Price N, Bellieny-Rabelo D, Zhu YK, Doudna JA. Birth of protein folds and functions in the virome. *Nature*. 2024/09/01 2024;633(8030):710-717. doi:10.1038/s41586-024-07809-y

34. Jones P, Binns D, Chang H-Y, et al. InterProScan 5: genome-scale protein function classification. *Bioinformatics*. 2014;30(9):1236-1240.

35. Marchler-Bauer A, Lu S, Anderson JB, et al. CDD: a Conserved Domain Database for the functional annotation of proteins. *Nucleic acids research*. 2010;39(suppl_1):D225-D229.

36. Haft DH, Selengut JD, Richter RA, Harkins D, Basu MK, Beck E. TIGRFAMs and genome properties in 2013. *Nucleic acids research*. 2012;41(D1):D387-D395.

37. Bateman A, Coin L, Durbin R, et al. The Pfam protein families database. *Nucleic acids research*. 2004;32(suppl_1):D138-D141.

38. Starr TN, Greaney AJ, Hannon WW, et al. Shifting mutational constraints in the SARS-CoV-2 receptor-binding domain during viral evolution. *Science*. 2022/07/22 2022;377(6604):420-424. doi:10.1126/science.abo7896

39. Welsh FC, Eguia RT, Lee JM, et al. Age-dependent heterogeneity in the antigenic effects of mutations to influenza hemagglutinin. *Cell Host & Microbe*. 2024;32(8):1397-1411. e11.

40. Bakhache W, Symonds-Orr W, McCormick L, Dolan PT. Deep mutation, insertion and deletion scanning across the Enterovirus A proteome reveals constraints shaping viral evolution. *Nature Microbiology*. 2025/01/01 2025;10(1):158-168. doi:10.1038/s41564-024-01871-y

41. Notin P, Kollasch A, Ritter D, et al. Proteingym: Large-scale benchmarks for protein fitness prediction and design. *Advances in Neural Information Processing Systems*. 2023;36:64331-64379.

42. Raybould MIJ, Kovaltsuk A, Marks C, Deane CM. CoV-AbDab: the coronavirus antibody database. *Bioinformatics*. 2021;37(5):734-735. doi:10.1093/bioinformatics/btaa739

43. Ye J, Ma N, Madden TL, Ostell JM. IgBLAST: an immunoglobulin variable domain sequence analysis tool. *Nucleic acids research*. 2013;41(W1):W34-W40.

44. Myung Y, Pires DEV, Ascher DB. CSM-AB: graph-based antibody–antigen binding affinity prediction and docking scoring function. *Bioinformatics*. 2022;38(4):1141-1143. doi:10.1093/bioinformatics/btab762

45. Yuan Y, Chen Q, Mao J, Li G, Pan X. DG-Affinity: predicting antigen–antibody affinity with language models from sequences. *BMC Bioinformatics*. 2023/11/13 2023;24(1):430. doi:10.1186/s12859-023-05562-z

46. Wang Y, Lv H, Teo QW, et al. An explainable language model for antibody specificity prediction using curated influenza hemagglutinin antibodies. *Immunity*. 2024;57(10):2453-2465. e7.

47. Wasdin PT, Johnson NV, Janke AK, et al. Generation of antigen-specific paired-chain antibodies using large language models. *Cell*. 2025;188(25):7206-7221.e16. doi:10.1016/j.cell.2025.10.006

48. Gao M, Skolnick J. Improved deep learning prediction of antigen–antibody interactions. *Proceedings of the National Academy of Sciences*. 2024;121(41):e2410529121. doi:doi:10.1073/pnas.2410529121

49. Gao M, Nakajima An D, Parks JM, Skolnick J. AF2Complex predicts direct physical interactions in multimeric proteins with deep learning. *Nature communications*. 2022;13(1):1744.

**II. Supplementary Figures**


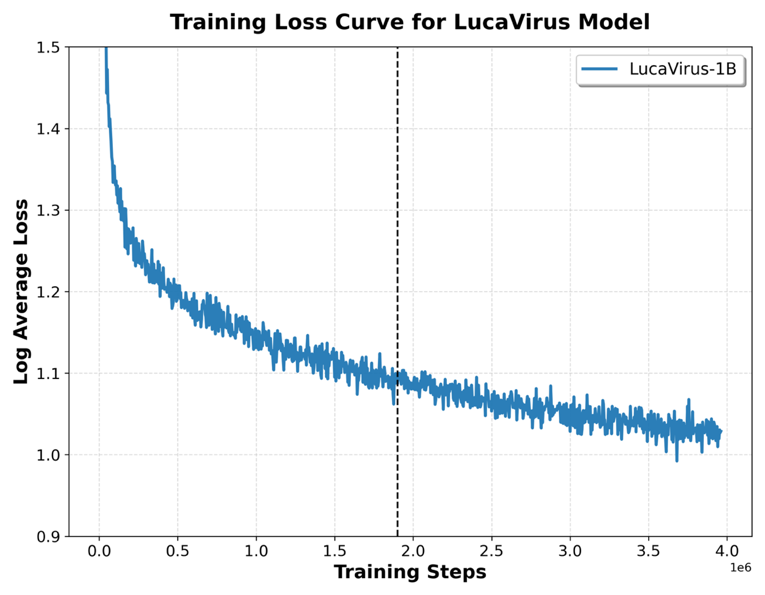
**Fig. S1. Pretraining loss curve for LucaVirus-1B model.** We trained LucaVirus using eight A100 GPUs on Alibaba Cloud for a total of 3,800,000 steps (approximately 1.9 epochs) over a duration of 70 days. 'Log Average Loss' refers to the average loss calculated every 4,000 steps. Dashed line indicates the end of first epoch.

**
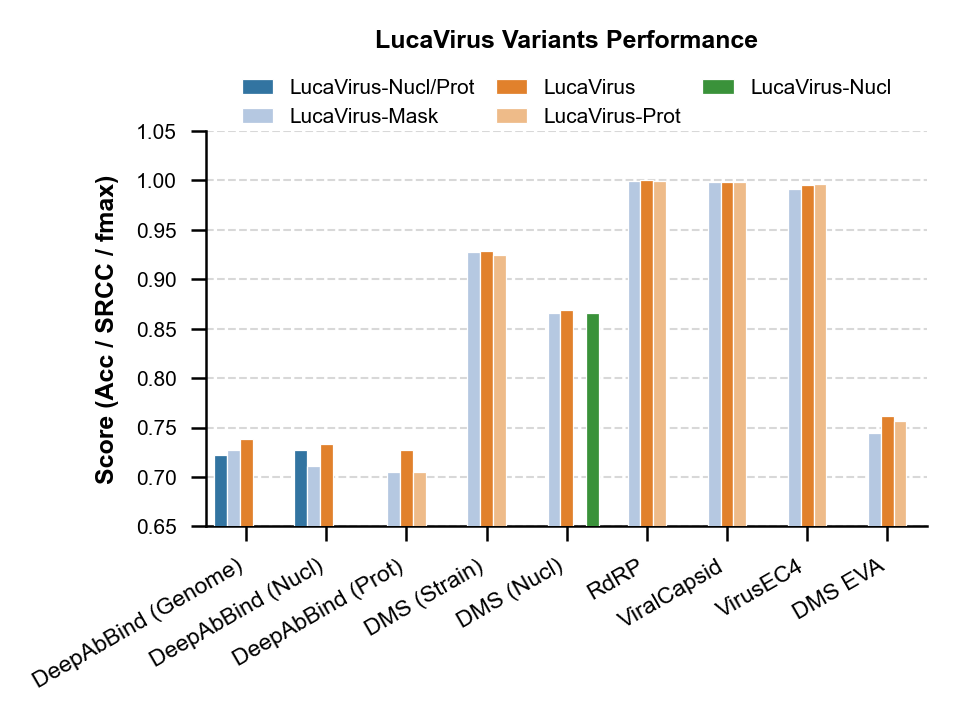
**

**Fig. S2. Ablation analysis of the LucaVirus architecture.**The impact of individual components is assessed across task types using standard metrics: accuracy for classification, Fmax for multi-label classification, and Spearman’s correlation coefficient (SRCC) for regression. Results identify critical elements for model efficacy. Refer to Table S3 for detailed data. Note: The antibody binding performance metrics in this table are based on all 98 antibodies, including those with weak binding affinity.

**Fig. S3. Generalization assessment of LucaVirus on a multi-modal benchmark of biological sequence tasks.** LucaVirus, trained on viral sequences, is compared to LucaOne on 10 original benchmark tasks spanning protein and nucleotide prediction. Performance metrics are classification accuracy and Spearman’s correlation for regression. The metrics for LucaOne were obtained at the 5,600,000-step checkpoint from LucaOne (20). The comparison underscores the model's broad applicability. Refer to Table S4 for a complete task breakdown.


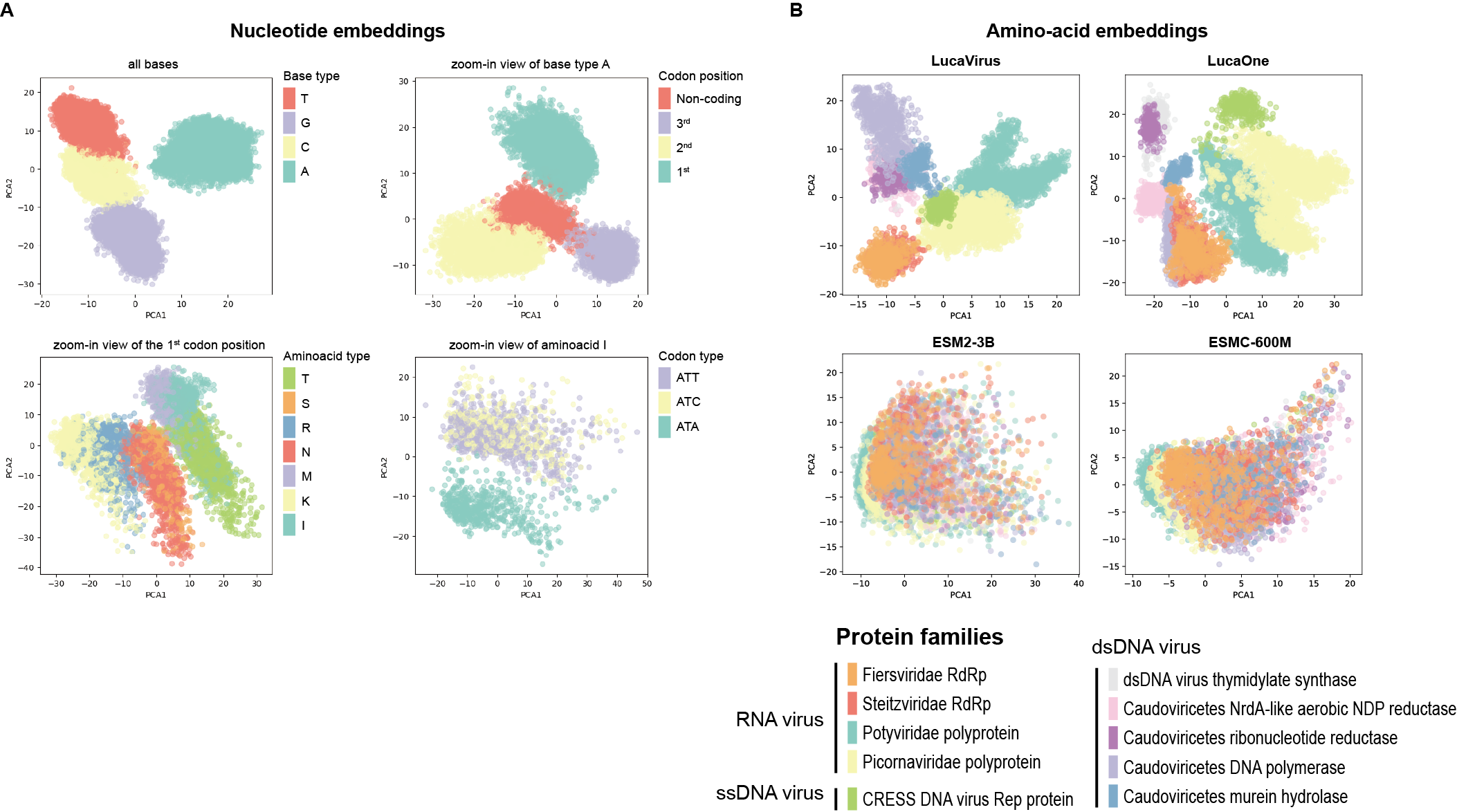
**Fig. S4. LucaVirus captures the relationship between nucleotide and protein sequences at single amino acid resolution.** (**A**) Nucleotide sequence features learned by LucaVirus. This panel visualizes the embedding matrix from 30 enterovirus reference genomes processed by LucaVirus, reduced via PCA. Each point represents a nucleotide token's embedding vector. The four sub-plots are: (i) PCA of all nucleotide tokens, colored by base type. (ii) PCA of adenine (A) tokens, colored by codon position. (iii) PCA of adenine tokens at the first codon position, colored by encoded amino acid. (iv) PCA of adenine tokens at the first position of codons encoding isoleucine (I), colored by codon type. (**B**) Viral protein feature spaces from different language models. This panel compares how LucaVirus, LucaOne, ESM2-3B, and ESMC-600M represent viral protein sequences from 100 proteins across 10 families. Embeddings are reduced via PCA, with points colored by protein family. Each point represents an amino acid token's embedding vector.

**
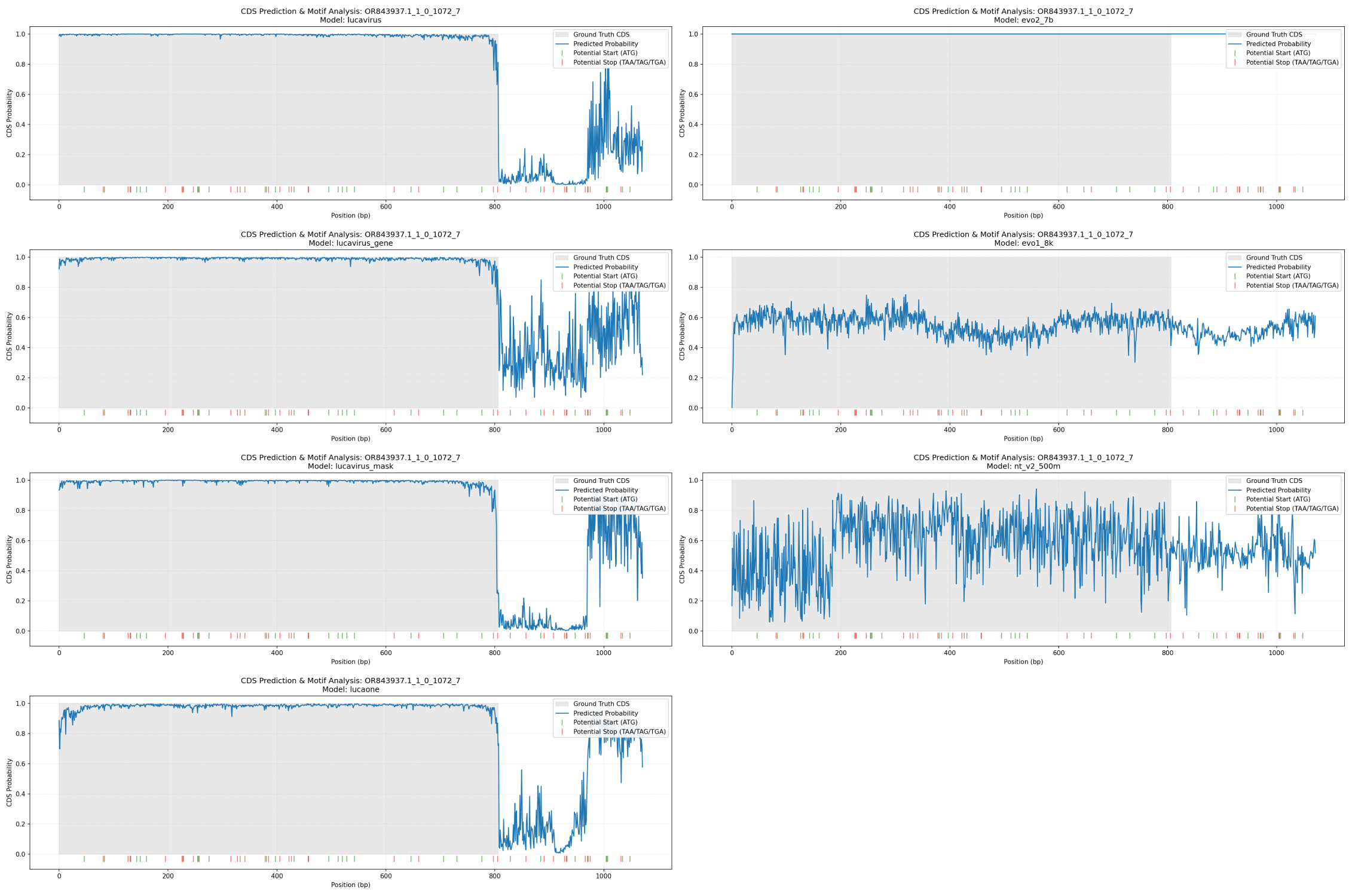
**

**Fig. S5. Comparative visualization of linear probe predictions for coding sequence (CDS) identification.** The plot depicts model outputs for the held-out exemplar OR843937.1. Gray shading denotes the ground-truth CDS region per NCBI annotation. The green and red line indicate location of start and stop codon respectively. The continuous blue trace for each model (LucaVirus, LucaVirus-Nucl, LucaVirus-Mask, LucaOne, Evo2, Evo1, Nucleotide Transformer) represents the per-position probability of being within a CDS, with a classification threshold at 0.5.

The results demonstrate that the Luca-family models successfully identified a non-complete coding region (clipped at the 5’-end, lacking a canonical start codon) while correctly ignoring other non-coding open reading frames (ORFs). Among these, LucaVirus achieved the highest accuracy and cleanest signal-to-noise ratio. In contrast, the Evo-family and Nucleotide Transformer models failed to accurately resolve the CDS boundaries or distinguish between coding and non-coding regions.

**
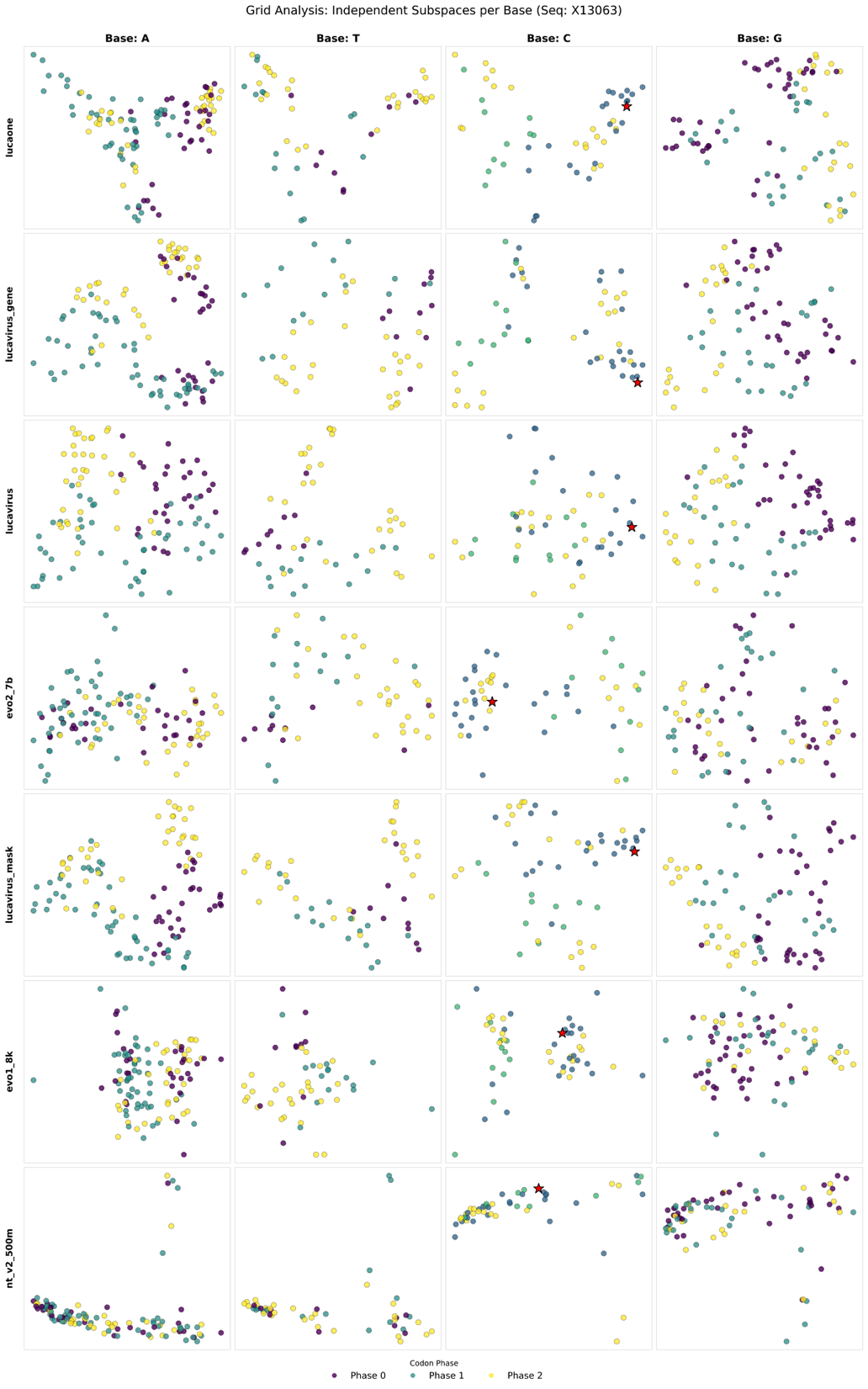
**

**Fig. S6. Comparative visualization of LLM embeddings near a programmed ribosomal frameshifting (PRF) site.** This grid analysis illustrates the embedding space for sequence X13063, specifically focusing on the region surrounding a -1 PRF event. The columns represent independent subspaces for each nucleotide base (A, T, C, G), while rows display results for different models: LucaOne, LucaVirus-Nucl, LucaVirus, Evo2-7b, LucaVirus-Mask, Evo1-8k, and NT-v2-500m.

Points are colored by codon phase (Phase 0: purple; Phase 1: teal; Phase 2: yellow), which represents the relative position of a base within a triplet codon. The red star indicates the programmed ribosomal frameshifting (slippery) site, where the ribosome shifts the reading frame by one base, thereby altering the subsequent codon phases.

The results demonstrate that the LucaVirus and related Luca-family embeddings effectively capture the local phase structure, shown by the distinct clustering of phases even across the frameshifting transition. In contrast, models like Evo2-7b, Evo1-8k, and NT-v2-500m exhibit significant mixing of phases in their embedding spaces, suggesting a failure to maintain a clear representation of the translational reading frame in the vicinity of non-canonical translation events.

**Fig. S7. Codon phase classification accuracy across a programmed ribosomal frameshifting (PRF) site.** Complementing the embedding analysis in Fig. S6, this figure displays the linear probe prediction results for sequence X13063. The vertical dashed line and arrow pinpoint the slippery site (at approximately 1380 bp) where a -1 PRF event occurs. Each horizontal track represents the performance of a specific model, with green segments indicating correct codon phase identification and red segments indicating misclassification.

The results reveal that LucaVirus and related Luca-family models maintain high classification accuracy both before and after the frameshifting event, demonstrating that their embeddings successfully capture the transition in the translational reading frame. In contrast, Evo2-7b, Evo1-8k, and NT-v2-500m exhibit a marked increase in errors (dense red regions) following the slippery site, suggesting that these models fail to adapt to non-canonical translational shifts. These findings highlight the superior capability of the Luca-family models in representing complex viral genomic features.


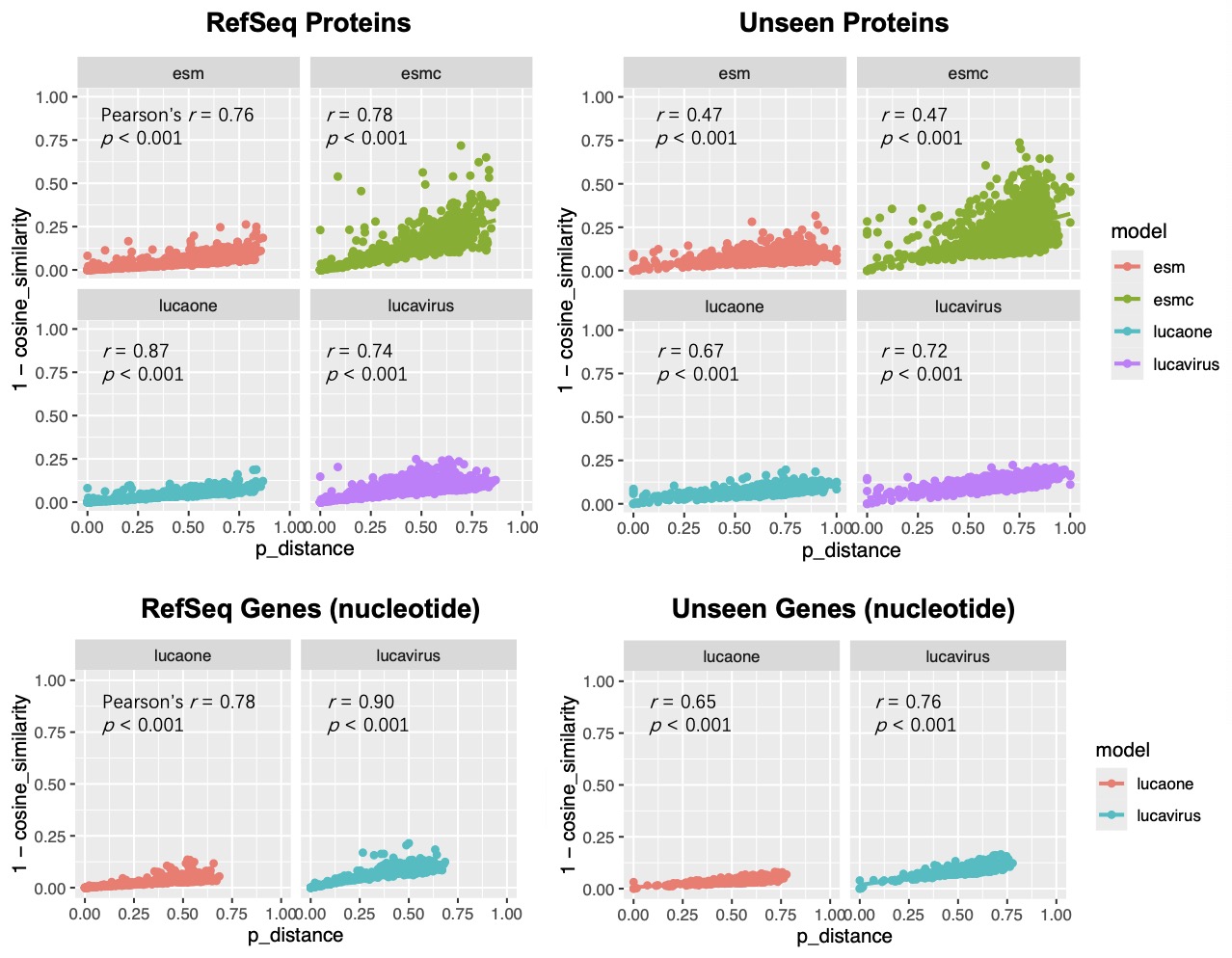
**Fig. S8. Correlation between the mean cosine distance of sequence embeddings and p-distance (1 – percent identity).** Among the popular LLMs tested, LucaVirus demonstrates the most robust and generalizable relationship with sequence divergence, maintaining strong performance even on previously unseen protein data sets. By contrast, the other three models show a marked drop in performance outside of their training data.

**Fig. S9. The pseudo-code for the LMAlign algorithm – an embedding-based Smith-Waterman alignment method.**

**
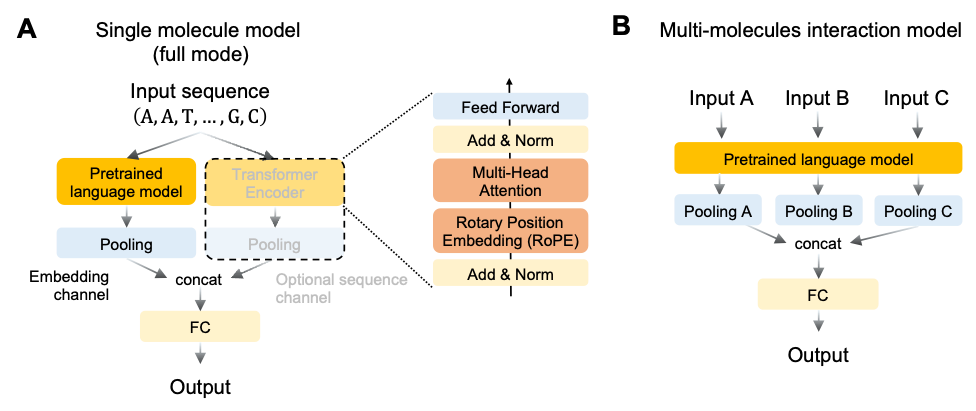
**

**Fig. S10. General framework for using pretrained language models for specific downstream tasks.** In all tasks examined in this study, the weights of the pretrained language model were kept frozen during downstream training.

**
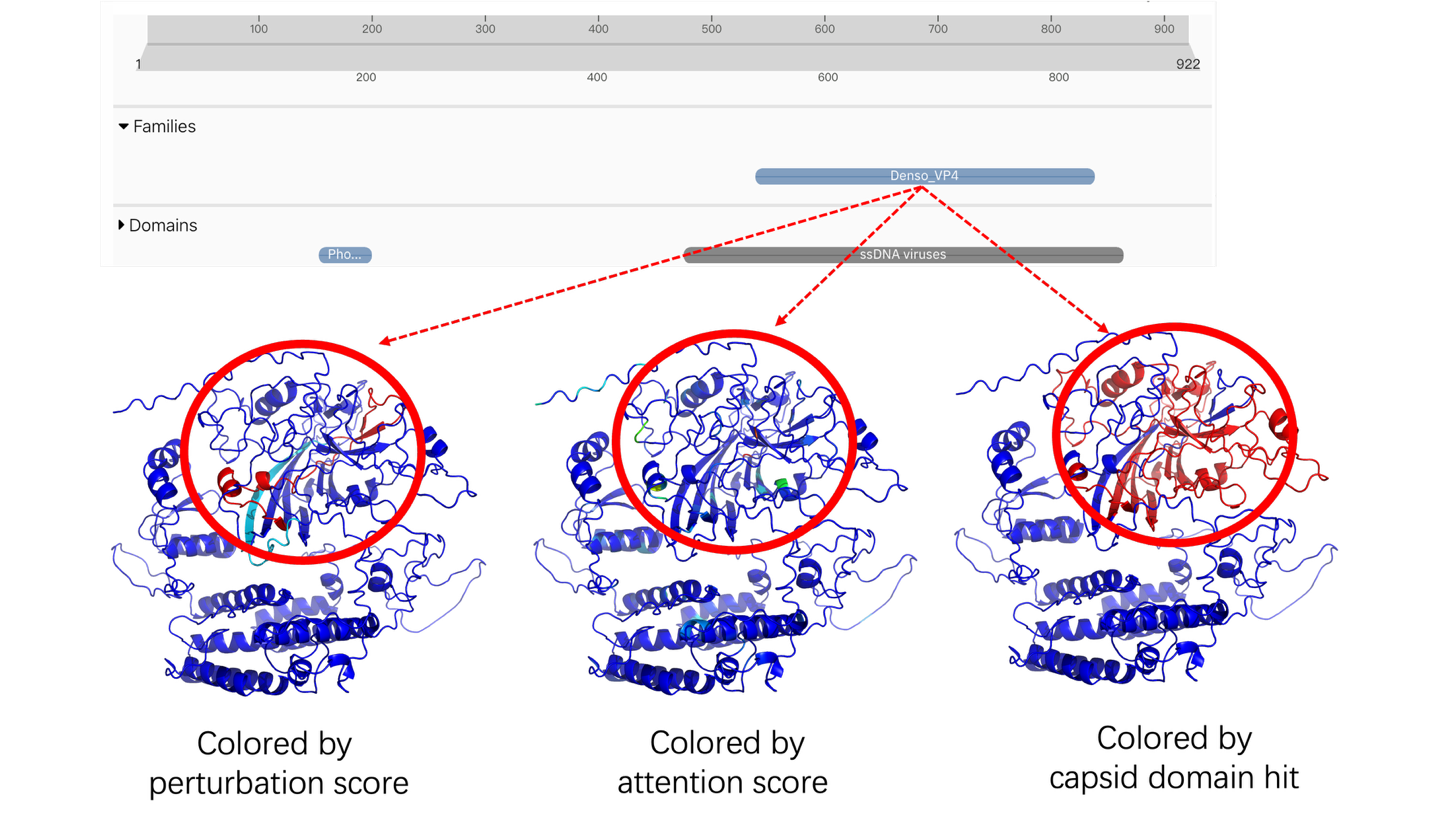
**

**Fig. S11. Visualization of the attention and perturbation maps for the LucaVirus capsid prediction model.** The perturbation score represents the z-score of the change in model output, calculated by sliding a 100-amino-acid (aa) mask with a 50-aa overlap across the wild-type sequence. This score reflects the key regions or domains that significantly impact the model's prediction. The 3D protein structure was predicted using ESMFold. The highlighted regions demonstrate that the model successfully focuses on the structural capsid domain within a polyprotein background, a finding consistently supported by both perturbation analysis and attention weights. The analysis is shown for sequence ID: lcl|ORF9_YC_k127_7119128:4293:1525.

**
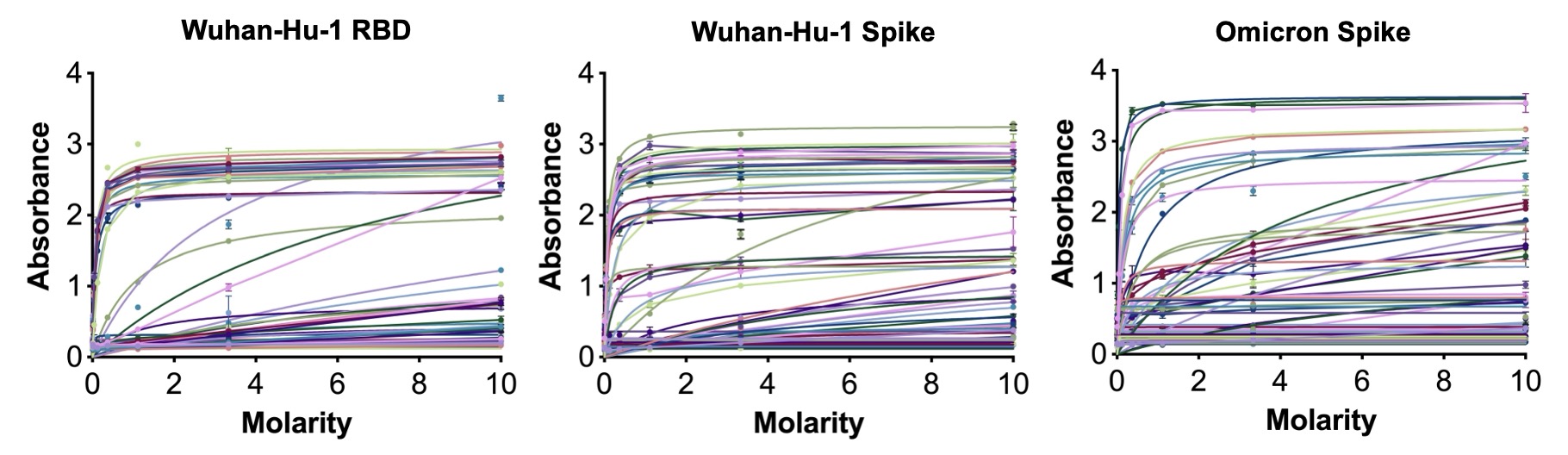
Fig. S12. Validation of antibody-antigen binding using ELISA.** We randomly selected 98 antibodies obtained through single B cell sequencing from convalescent COVID-19 patients in our previous study. ELISA assays were performed to assess the binding of these antibodies to the receptor-binding domain (RBD) and full-length Spike protein of the Wuhan-Hu-1 strain, as well as the full-length Spike protein of the Omicron variant.

**III. Supplementary Tables**

**Table S1. Composition of pre-training data set**

| **Source** | **Type** | **Token counts** | **Sequence counts** | **Seq-level annotation** | **Span-level annotation** |
| --- | --- | --- | --- | --- | --- |
| NCBI Virus | Nucleotide | 2,322,433,600 | 3,233,945 | taxonomy | CDS |
| Wolf et al. (ref *22*) | Nucleotide | 1,121,406,280 | 1,875,616 | n/a | n/a |
| Gregory et al. (ref *24*) | Nucleotide | 1,465,432,141 | 1,875,225 | n/a | n/a |
| Hou et al. (ref 9) | Nucleotide | 891,253,024 | 373,079 | n/a | n/a |
| GPD (ref *21*) | Nucleotide | 349,588,480 | 2,910,001 | n/a | n/a |
| Neri et al. (ref *20*) | Nucleotide | 393,776,802 | 168,727 | n/a | n/a |
| Zayed et al. (ref *23*) | Nucleotide | 28,931,582 | 29,112 | n/a | n/a |
| UniProt-Trembl | Protein | 1,627,140,177 | 5,164,087 | taxonomy/keywords | Super family/domain/site |
| UniProt-SwissProt | Protein | 7,779,199 | 17,212 | taxonomy/keywords | Super family/domain/site |
| UniProt-UniRef50 | Protein | 141,972,000 | 605,379 | taxonomy/keywords | Super family/domain/site |
| ColabFold-envdb | Protein | 75,973,285 | 282,640 | taxonomy | n/a |

**Table S2. Model configuration of six LucaVirus pre-trained variants.**

| **Pretrained model** | **Parameters** | **Embedding dimension** | **Pretraining tasks** | **Modality** |
| --- | --- | --- | --- | --- |
| LucaVirus-Large | 1B | 2560 | MLM + semi-supervised tasks | Protein/Nucleotide |
| LucaVirus-Base | 112M | 256 | MLM + semi-supervised tasks | Protein/Nucleotide |
| LucaVirus-Small | 62M | 128 | MLM + semi-supervised tasks | Protein/Nucleotide |
| LucaVirus-Mask | 1B | 2560 | MLM only | Protein/Nucleotide |
| LucaVirus-Prot | 1B | 2560 | MLM + semi-supervised tasks | Protein |
| LucaVirus-Nucl | 1B | 2560 | MLM + semi-supervised tasks | Nucleotide |

**Table S3. Detailed performance metrics for model ablation studies.**

| **Task** | **Model** | **Classification metrics** | | | | | **Multi-label metrics** | | **Regression metrics** | |
| --- | --- | --- | --- | --- | --- | --- | --- | --- | --- | --- |
|  |  | **Acc** | **F1** | **AUC** | **PR-AUC** | **MCC** | **Jaccard** | **Fmax** | **Spearman** | **Pearson** |
| DeepAbBindv2  (genome) | LucaVirus-Nucl/Prot | 0.7222 | 0.5192 | **0.6950** | 0.5801 | 0.3464 |  |  |  |  |
|  | LucaVirus-Mask | 0.7277 | 0.5242 | 0.6648 | **0.5862** | 0.3589 |  |  |  |  |
|  | LucaVirus | **0.7388** | **0.5765** | 0.6913 | 0.5529 | **0.3972** |  |  |  |  |
| DeepAbBindv2  (nucl) | LucaVirus-Nucl/Prot | 0.7277 | 0.5148 | 0.6800 | 0.5801 | 0.3566 |  |  |  |  |
|  | LucaVirus-Mask | 0.7111 | 0.5357 | 0.6319 | 0.5113 | 0.3335 |  |  |  |  |
|  | LucaVirus | **0.7333** | **0.5385** | **0.6951** | **0.6024** | **0.3741** |  |  |  |  |
| DeepAbBindv2  (original) | LucaVirus-Prot | 0.7055 | 0.5046 | 0.6755 | 0.5918 | 0.3104 |  |  |  |  |
|  | LucaVirus-Mask | 0.7055 | 0.4952 | 0.6603 | 0.5179 | 0.3067 |  |  |  |  |
|  | LucaVirus | **0.7278** | **0.5812** | **0.7124** | **0.6107** | **0.3821** |  |  |  |  |
| DMS_RBD  (original) | LucaVirus-Prot |  |  |  |  |  |  |  | 0.9243 | 0.9470 |
|  | LucaVirus-Mask |  |  |  |  |  |  |  | 0.9277 | 0.9440 |
|  | LucaVirus |  |  |  |  |  |  |  | **0.9291** | **0.9477** |
| DMS_RBD  (nucl) | LucaVirus-Nucl |  |  |  |  |  |  |  | 0.8663 | 0.9193 |
|  | LucaVirus-Mask |  |  |  |  |  |  |  | 0.8660 | 0.9212 |
|  | LucaVirus |  |  |  |  |  |  |  | **0.8692** | **0.9247** |
| RdRP | LucaVirus-Prot | 0.9999 | 0.9981 | 0.9999 | 0.9999 | 0.9981 |  |  |  |  |
|  | LucaVirus-Mask | 0.9997 | 0.9944 | 0.9998 | 0.9983 | 0.9943 |  |  |  |  |
|  | LucaVirus | **1.0** | **1.0** | **1.0** | **1.0** | **1.0** |  |  |  |  |
| ViralCapsid | LucaVirus-Prot | **0.9988** | **0.9988** | **0.9997** | **0.9996** | **0.9976** |  |  |  |  |
|  | LucaVirus-Mask | 0.9983 | 0.9983 | 0.9996 | 0.9992 | 0.9966 |  |  |  |  |
|  | LucaVirus | 0.9985 | 0.9985 | 0.9996 | 0.9993 | 0.9971 |  |  |  |  |
| VirusEC4 | LucaVirus-Prot |  |  |  |  |  | **0.9929** | **0.9965** |  |  |
|  | LucaVirus-Mask |  |  |  |  |  | 0.9822 | 0.9912 |  |  |
|  | LucaVirus |  |  |  |  |  | 0.9907 | 0.9957 |  |  |
| DMS_EVA | LucaVirus-Mask |  |  |  |  |  |  |  | 0.7445 | 0.7820 |
|  | LucaVirus-Prot |  |  |  |  |  |  |  | 0.7562 | 0.7912 |
|  | LucaVirus |  |  |  |  |  |  |  | **0.7616** | **0.7984** |

Note: The antibody binding performance metrics in this table are based on all 98 antibodies, including those with weak binding affinity.

**Table S4. Performance comparison of LucaVirus and LucaOne on ten general tasks.**

| **Task** | **Model** | **Acc** | **F1** | **PR-AUC** | **Spearman** |
| --- | --- | --- | --- | --- | --- |
| CentralDogma | LucaOne | **0.8453** | **0.7392** | **0.8617** | **/** |
|  | LucaVirus | 0.7537 | 0.592758 | 0.680429 | / |
| SupKTax | LucaOne | 0.947 | 0.9467 | 0.9591 | / |
|  | LucaVirus | **0.949** | **0.947768** | **0.959657** | **/** |
| GenusTax | LucaOne | 0.817 | 0.8069 | 0.8633 | / |
|  | LucaVirus | **0.863** | **0.857885** | **0.901058** | **/** |
| SpeciesTax | LucaOne | 0.75 | 0.7345 | 0.7927 | / |
|  | LucaVirus | **0.784** | **0.769504** | 0.782852 | / |
| ncRNAFam | LucaOne | **0.9864** | 0.9117 | **0.9622** | **/** |
|  | LucaVirus | 0.982559 | 0.911888 | 0.952142 | / |
| ProtLoc | LucaOne | **0.9452** | **0.9378** | 0.9692 | / |
|  | LucaVirus | 0.93634 | 0.93306 | **0.969878** | **/** |
| ProtStab | LucaOne | **/** | / | / | **0.7718** |
|  | LucaVirus | / | / | / | 0.743177 |
| InfA | LucaOne | 1.0 | 1.0 | 1.0 | / |
|  | LucaVirus | 1.0 | 1.0 | 1.0 | / |
| PPI | LucaOne | 0.9774 | 0.9799 | 0.9922 | / |
|  | LucaVirus | **0.979394** | **0.981727** | **0.992441** | **/** |
| ncRPI | LucaOne | **0.9479** | **0.947** | 0.9781 | / |
|  | LucaVirus | 0.947887 | 0.946801 | **0.978143** | **/** |

**Table S5. PERMANOVA analysis of embedding spaces for differentiating sequence features.**Statistical testing of model embeddings' capacity to separate sequences by three distinct biological features: amino acid identity, codon phase, and codon type.

| **Variable** | **R2** | **R2_Adj** | **P-value** | **Model_Name** |
| --- | --- | --- | --- | --- |
| base | 0.20229846 | 0.20217878 | 0.001 | LucaOne |
| codon_phase | 0.04295975 | 0.04286403 | 0.001 | LucaOne |
| aminoacid | 0.10865857 | 0.10772163 | 0.001 | LucaOne |
| codon | 0.14390163 | 0.14115318 | 0.001 | LucaOne |
| Full_Model | 0.39108998 | 0.38833736 | 0.001 | LucaOne |
| base | 0.08336025 | 0.08322273 | 0.001 | LucaVirus |
| codon_phase | 0.02812825 | 0.02803104 | 0.001 | LucaVirus |
| aminoacid | 0.056764 | 0.05577251 | 0.001 | LucaVirus |
| codon | 0.07781359 | 0.07485297 | 0.001 | LucaVirus |
| Full_Model | 0.20462631 | 0.20103077 | 0.001 | LucaVirus |
| base | 0.11903172 | 0.11889955 | 0.001 | LucaVirus-Nucl |
| codon_phase | 0.03722265 | 0.03712636 | 0.001 | LucaVirus-Nucl |
| aminoacid | 0.07113074 | 0.07015435 | 0.001 | LucaVirus-Nucl |
| codon | 0.10059895 | 0.09771149 | 0.001 | LucaVirus-Nucl |
| Full_Model | 0.27487034 | 0.27159234 | 0.001 | LucaVirus-Nucl |
| base | 0.09953561 | 0.09940051 | 0.001 | LucaVirus-Mask |
| codon_phase | 0.02675398 | 0.02665664 | 0.001 | LucaVirus-Mask |
| aminoacid | 0.06487574 | 0.06389278 | 0.001 | LucaVirus-Mask |
| codon | 0.08852835 | 0.08560213 | 0.001 | LucaVirus-Mask |
| Full_Model | 0.22786513 | 0.22437464 | 0.001 | LucaVirus-Mask |
| base | 0.03261618 | 0.03247104 | 0.001 | EVO1-8K |
| codon_phase | 0.04218625 | 0.04209045 | 0.001 | EVO1-8K |
| aminoacid | 0.0560819 | 0.05508969 | 0.001 | EVO1-8K |
| codon | 0.07264765 | 0.06967044 | 0.001 | EVO1-8K |
| Full_Model | 0.16064847 | 0.15685412 | 0.001 | EVO1-8K |
| base | 0.03964316 | 0.03949908 | 0.001 | EVO2-7B |
| codon_phase | 0.03088893 | 0.030792 | 0.001 | EVO2-7B |
| aminoacid | 0.03546567 | 0.03445179 | 0.001 | EVO2-7B |
| codon | 0.05147731 | 0.04843214 | 0.001 | EVO2-7B |
| Full_Model | 0.13530725 | 0.13139835 | 0.001 | EVO2-7B |
| base | 0.00279747 | 0.00264786 | 0.001 | NT-V2-500M |
| codon_phase | 6.44E-05 | -3.56E-05 | 0.49 | NT-V2-500M |
| aminoacid | 0.00374634 | 0.00269913 | 0.001 | NT-V2-500M |
| codon | 0.01799032 | 0.01483764 | 0.001 | NT-V2-500M |
| Full_Model | 0.02111109 | 0.01668596 | 0.001 | NT-V2-500M |

**Table S6. Linear probing for genomic feature classification from model embeddings.** Reports classification performance (e.g., accuracy) of simple linear models trained to predict four distinct genomic features from frozen embeddings: CDS vs. non-CDS regions, amino acid identity, codon phase (reading frame), and codon type.

|  | **Acc** |  |  |  |  |  | **F1** |  |  |  |  |  |
| --- | --- | --- | --- | --- | --- | --- | --- | --- | --- | --- | --- | --- |
| **Model** | **CDS** | **base** | **phase** | **codon** | **aa** | **RBFS_phase** | **CDS** | **base** | **phase** | **codon** | **aa** | **RBFS_phase** |
| LucaVirus | 0.9380 | 1.0000 | 0.9163 | 0.8794 | 0.8642 | 0.7338 | 0.9380 | 1.0000 | 0.9163 | 0.8768 | 0.8616 | 0.7366 |
| LucaVirus-Nucl | 0.9330 | 1.0000 | 0.9113 | 0.8675 | 0.8437 | 0.7015 | 0.9330 | 1.0000 | 0.9113 | 0.8649 | 0.8409 | 0.6980 |
| LucaVirus-Mask | 0.9240 | 1.0000 | 0.9127 | 0.8811 | 0.8726 | 0.6915 | 0.9240 | 1.0000 | 0.9127 | 0.8782 | 0.8702 | 0.6919 |
| LucaOne | 0.9295 | 1.0000 | 0.9013 | 0.8637 | 0.8585 | 0.6642 | 0.9294 | 1.0000 | 0.9014 | 0.8596 | 0.8560 | 0.6642 |
| Evo-1-8k | 0.6285 | 0.3862 | 0.5580 | 0.0455 | 0.1020 | 0.4030 | 0.6284 | 0.3432 | 0.5394 | 0.0278 | 0.0784 | 0.4002 |
| Evo2-7B | 0.7425 | 0.9695 | 0.8033 | 0.1053 | 0.1863 | 0.6169 | 0.7402 | 0.9696 | 0.8041 | 0.0887 | 0.1509 | 0.6198 |
| NT-v2-500M | 0.7465 | 0.3098 | 0.3363 | 0.0384 | 0.0785 | 0.3234 | 0.7463 | 0.3081 | 0.3316 | 0.0322 | 0.0669 | 0.2891 |

**Table S7. Correlation between language model embeddings and genetic divergence of protein sequences.** Best metrics are colored red.

|  | **Metrics** | **Unseen Proteins** | | | | **RefSeq Proteins** | | | |
| --- | --- | --- | --- | --- | --- | --- | --- | --- | --- |
|  |  | **LucaVirus** | **LucaOne** | **ESM2** | **ESMC** | **LucaVirus** | **LucaOne** | **ESM2** | **ESMC** |
| Pearson Correlation | Cosine distance vs. *p* distance | 0.71788789 | 0.67292459 | 0.46732339 | 0.46945701 | 0.73814079 | 0.86718098 | 0.75981986 | 0.77930478 |
|  | *P* value | 0 | 0 | 0 | 0 | 0 | 0 | 0 | 0 |
|  | Euclidean distance vs. *p* distance | 0.77199537 | 0.68346771 | 0.55707626 | 0.55494094 | 0.88842785 | 0.89616379 | 0.89246256 | 0.89877686 |
|  | *P* value | 0 | 0 | 0 | 0 | 0 | 0 | 0 | 0 |
|  | Cosine distance vs. phylogenetic distance* | 0.52350999 | 0.53489835 | 0.37349514 | 0.39681662 | 0.57705498 | 0.74245948 | 0.65126758 | 0.66326018 |
|  | *P* value | 0 | 0 | 0 | 0 | 0 | 0 | 0 | 0 |
|  | Euclidean distance vs. phylogenetic distance | 0.54526862 | 0.51891972 | 0.42307802 | 0.44204553 | 0.678809 | 0.71812782 | 0.71949099 | 0.72971626 |
|  | *P* value | 0 | 0 | 0 | 0 | 0 | 0 | 0 | 0 |
| Spearman Correlation | Cosine distance vs. *p* distance | 0.69488777 | 0.69120009 | 0.53346517 | 0.52730391 | 0.71814951 | 0.86579372 | 0.86433049 | 0.86534489 |
|  | *P* value | 0 | 0 | 0 | 0 | 0 | 0 | 0 | 0 |
|  | Euclidean distance vs. *p* distance | 0.74883721 | 0.66831573 | 0.55415761 | 0.58649128 | 0.82507138 | 0.84648674 | 0.87746318 | 0.88903646 |
|  | *P* value | 0 | 0 | 0 | 0 | 0 | 0 | 0 | 0 |
|  | Cosine distance vs. phylogenetic distance | 0.52966593 | 0.56079703 | 0.41000241 | 0.41266814 | 0.70513822 | 0.849783 | 0.82559709 | 0.82786262 |
|  | *P* value | 0 | 0 | 0 | 0 | 0 | 0 | 0 | 0 |
|  | Euclidean distance vs. phylogenetic distance | 0.57467934 | 0.5362321 | 0.42339089 | 0.45910491 | 0.80493673 | 0.8389194 | 0.8387283 | 0.85415401 |
|  | *P* value | 0 | 0 | 0 | 0 | 0 | 0 | 0 | 0 |

*Phylogenetic distances were computed from a maximum likelihood (ML) phylogenetic tree and represent the estimated number of substitutions per site between pairs of homologous sequences.

**Table S8. Correlation between language model embeddings and genetic divergence of nucleotide sequences.** Best metrics are colored red.

|  | **Metrics** | **Unseen Genes (nucleotide)** | | **RefSeq Genes (nucleotide)** | |
| --- | --- | --- | --- | --- | --- |
|  |  | **LucaVirus** | **LucaOne** | **LucaVirus** | **LucaOne** |
| Pearson Correlation | Cosine distance vs. *p* distance | 0.76357767 | 0.64819183 | 0.90289818 | 0.77512818 |
|  | *P* value | 0 | 0 | 0 | 0 |
|  | Euclidean distance vs. *p* distance | 0.82627862 | 0.71496931 | 0.93553203 | 0.90442508 |
|  | *P* value | 0 | 0 | 0 | 0 |
|  | Cosine distance vs. phylogenetic distance* | 0.53775195 | 0.48494253 | 0.67405342 | 0.56478566 |
|  | *P* value | 0 | 0 | 0 | 0 |
|  | Euclidean distance vs. phylogenetic distance | 0.56996224 | 0.51259255 | 0.67548841 | 0.63976091 |
|  | *P* value | 0 | 0 | 0 | 0 |
| Spearman Correlation | Cosine distance vs. *p* distance | 0.72093615 | 0.6149491 | 0.84638142 | 0.81946972 |
|  | *P* value | 0 | 0 | 0 | 0 |
|  | Euclidean distance vs. *p* distance | 0.78376811 | 0.64234757 | 0.89161081 | 0.83500575 |
|  | *P* value | 0 | 0 | 0 | 0 |
|  | Cosine distance vs. phylogenetic distance | 0.55254023 | 0.48869635 | 0.8005068 | 0.76488604 |
|  | *P* value | 0 | 0 | 0 | 0 |
|  | Euclidean distance vs. phylogenetic distance | 0.61729172 | 0.51256412 | 0.8488809 | 0.78273279 |
|  | *P* value | 0 | 0 | 0 | 0 |

*Phylogenetic distances were computed from a maximum likelihood (ML) phylogenetic tree and represent the estimated number of substitutions per site between pairs of homologous sequences.

**Table S9. Performance comparison of different LLMs on classification tasks.**

| Tasks | Method | Acc | F1 | AUC | PR-AUC | MCC |
| --- | --- | --- | --- | --- | --- | --- |
| DeepAbBindv2_genome | ESM2+DNABert2 | 0.733333 | 0.529412 | 0.665801 | 0.580468 | 0.371831 |
|  | ESM2+NT | 0.716667 | 0.548673 | 0.672499 | **0.608799** | 0.348529 |
|  | LucaOne | 0.733333 | 0.555556 | **0.702023** | 0.606699 | 0.37943 |
|  | LucaVirus | **0.738889** | **0.576577** | 0.691361 | 0.552956 | **0.397208** |
| DeepAbBindv2_nucl | ESM2+DNABert2 | 0.677778 | 0.462963 | 0.658488 | 0.509923 | 0.245408 |
|  | ESM2+NT | 0.672222 | 0.504202 | 0.656985 | 0.53887 | 0.260546 |
|  | LucaOne | 0.694444 | 0.495413 | 0.642701 | 0.492226 | 0.287761 |
|  | LucaVirus | **0.733333** | **0.538462** | **0.695052** | **0.60243** | **0.37409** |
| DeepAbBindv2_original | ESMC | 0.688889 | 0.517241 | 0.663546 | 0.601243 | 0.290843 |
|  | ESM2 | 0.7 | 0.517857 | 0.683024 | 0.560254 | 0.307427 |
|  | LucaOne | 0.705556 | 0.522523 | 0.687534 | 0.550662 | 0.318408 |
|  | LucaVirus | **0.727778** | **0.581197** | **0.712411** | **0.610665** | **0.382117** |
| Phage_PVP | ESMC | 0.989348 | 0.953226 | 0.995852 | 0.963023 | 0.978936 |
|  | ESM2 | **0.991209** | **0.958569** | 0.995687 | **0.965605** | **0.982621** |
|  | LucaOne | 0.987238 | 0.945649 | **0.996022** | 0.953041 | 0.974751 |
|  | LucaVirus | 0.987176 | 0.946065 | 0.994515 | 0.947646 | 0.974588 |
| RdRP | ESMC | 0.999865 | 0.99723 | 0.999999 | 0.99998 | 0.997165 |
|  | ESM2 | 0.999865 | 0.99723 | 0.999999 | 0.999976 | 0.997165 |
|  | LucaOne | 0.99991 | 0.998145 | 0.999997 | 0.999884 | 0.998101 |
|  | LucaVirus | **1.0** | **1.0** | **1.0** | **1.0** | **1.0** |
| ViralCapsid | ESMC | 0.997739 | 0.99774 | 0.999488 | 0.998993 | 0.995479 |
|  | ESM2 | 0.998308 | 0.998308 | 0.999639 | 0.999299 | 0.996617 |
|  | LucaOne | 0.998293 | 0.998293 | 0.999646 | 0.999341 | 0.996586 |
|  | LucaVirus | **0.998554** | **0.998554** | **0.999656** | **0.999371** | **0.997109** |

**Table S10. Performance comparison of different LLMs on multi-label classification task.**

| Tasks | Method | Jaccard | fmax |
| --- | --- | --- | --- |
| VirusEC4 | ESMC | 0.990217 | 0.995257 |
|  | ESM2 | **0.993335** | **0.996788** |
|  | LucaOne | 0.991878 | 0.99632 |
|  | LucaVirus | 0.990781 | 0.995724 |

**Table S11. Performance comparison of different LLMs on regression tasks.**

| Tasks | Method | SRCC (Spearman's correlation coefficient) | PRCC (Pearson correlation coefficient) |
| --- | --- | --- | --- |
| DMS_EVA | ESMC-600M | 0.683563 | 0.720946 |
|  | ESM2-3B | 0.758408 | 0.791698 |
|  | LucaOne | 0.753631 | 0.785300 |
|  | LucaVirus | **0.761608** | **0.798425** |
| DMS_Bind_Reps_Strain | ESMC-600M | 0.706095 | 0.769857 |
|  | ESM2-3B | 0.837381 | 0.887300 |
|  | LucaOne | 0.901307 | 0.943520 |
|  | LucaVirus | **0.929127** | **0.947747** |
| DMS_Bind_Reps_Strain_Nucl | DNABert2 | 0.794032 | 0.870466 |
|  | NT-2.5B-MultiSpecies | 0.846765 | 0.909852 |
|  | LucaOne | 0.856909 | 0.924048 |
|  | LucaVirus | **0.869273** | **0.924707** |
| HK19_immune_escape_per_serum | ESMC-600M | 0.239878 | 0.231521 |
|  | ESM2-3B | 0.512224 | 0.612066 |
|  | LucaOne | 0.502234 | 0.642996 |
|  | LucaVirus | **0.537990** | **0.723446** |

**Table S12. Confusion matrix of the wet-lab validated antibodies prediction by LucaVirus model (weak binding (+/++) excluded).**

| **Variant: Wuhan-Hu-1** | | Prediction | |
| --- | --- | --- | --- |
|  |  | Neg. | Pos. |
| Ground Truth | Neg. | 53 | 6 |
|  | Pos. | 6 | 13 |
| **Variant: Omicron** | | Prediction | |
|  |  | Neg. | Pos. |
| Ground Truth | Neg. | 71 | 4 |
|  | Pos. | 3 | 7 |

**Table S13. Wet-lab validated antibodies information.**

| **Name** | **Published Name** | **cell_id** | **WT Spike binding** | **Omicron Spike binding** |
| --- | --- | --- | --- | --- |
| I12 | CAV-C12 | I3_GTAGTCAAGGTGCAAC_1 | + | + |
| I14-2 | CAV-C14 | I4_CGTCAGGAGTGACTCT_1 | + | - |
| I17 | CAV-C17 | I1_CGGAGTCAGAAGGGTA_1 | + | - |
| I23 | CAV-C23 | I5_TTTCCTCCATAGACTC_1 | - | - |
| I28 | CAV-C28 | I5_GGATTACTCCTAGGGC_1 | - | - |
| I31 | CAV-C31 | I5_TCAGCTCTCGCTTAGA_1 | - | - |
| I35 | CAV-C35 | I1_CGGACTGGTATTAGCC_1 | - | - |
| I39 | CAV-C39 | SCoV1-M0_AGCTTGAAGGAGTTTA_1 | +++ | - |
| I40 | CAV-C40 | SCoV13-M0_AAACGGGTCTGCGGCA_1 | + | + |
| I44 | CAV-C44 | K1_CCACTACGTCTCTTTA_1 | +++ | +++ |
| I47 | CAV-C47 | K1_CTTAGGAGTTTAGCTG_1 | +++ | ++ |
| I59 | CAV-C59 | I7_GAACATCCAAGCGATG_1 | + | - |
| I60 | CAV-C60 | I8_ACGCCAGTCCCTAATT_1 | - | - |
| I61 | CAV-C61 | I8_TCAATCTGTGTGGCTC_1 | +++ | +++ |
| I65 | CAV-C65 | I7_CGCTGGAGTCGAAAGC_1 | +++ | + |
| I68 | CAV-C68 | I9_AACCGCGTCTGCAGTA_1 | +++ | +++ |
| I74 | CAV-C74 | I10_TGCTGCTGTATATCCG_1 | +++ | +++ |
| I75 | CAV-C75 | I10_GCGCGATAGCGAAGGG_1 | +++ | +++ |
| I76 | CAV-C76 | I9_CTACACCTCGAACGGA_1 | +++ | +++ |
| I77 | CAV-C77 | I9_GTCGTAAAGACCTAGG_1 | +++ | ++ |
| I79 | CAV-C79 | I10_CACACTCGTGAGGGAG_1 | +++ | +++ |
| I90 | CAV-C90 | SCoV11-M0_GACACGCTCCTAGAAC_1 | + | - |
| I91 | CAV-C91 | SCoV1-M0_TGATTTCTCATCGCTC_1 | - | - |
| I95 | CAV-C95 | I1_GGATTACCAAGCTGGA_1 | ++ | + |
| I96 | CAV-C96 | I2_GTACTCCCAGCCTTGG_1 | ++ | - |
| I97 | CAV-C97 | I3_TGCGGGTGTGTAAGTA_1 | ++ | + |
| I98 | CAV-C98 | I7_CACAGGCTCTTAGAGC_1 | + | - |
| CD26 | CAV-C26 | DG15-P1-B2 | + | - |
| CD155 | CAV-C155 | SG04-P1-H9 | + | - |
| CD217 | CAV-C217 | DG18-P2-D7 | +++ | - |
| CD218 | CAV-C218 | DG18-P2-E4 | +++ | - |
| CD219 | CAV-C219 | SG04-P2-A11 | + | + |
| CD236 | CAV-C236 | SC50-P1-A5 | +++ | +++ |
| CD241 | CAV-C241 | WH46-C1 | + | + |
| CD286 | CAV-C286 | WH19-P1-C4 | +++ | - |
| CD311 | CAV-C311 | DG29-P2-D6 | +++ | ++ |
| CD327 | CAV-C327 | SC2-074-P3-H3 | +++ | +++ |
| CD329 | CAV-C329 | SC2-074-P3-C4 | + | - |
| CD339 | CAV-C339 | WH28-P1-F5 | +++ | +++ |
| CD354 | CAV-C354 | WH2-1B-P3P5-C2 | ++ | - |
| CD355 | CAV-C355 | WH2-1B-P3P5-E11 | + | + |
| CD358 | CAV-C358 | WH2-1B-P3P5-D7 | - | - |
| CD362 | CAV-C362 | WH2-14-P2-A6 | + | - |
| CD368 | CAV-C368 | WH2-14-P2-E3 | + | - |
| CD380 | CAV-C380 | WH2-23-PC-A4 | ++ | + |
| CD385 | CAV-C385 | WH35-P2-H3 | +++ | ++ |
| I14-1 |  | I4_CGTCAGGAGTGACTCT_1 | - | - |
| I20 |  | K2_ACCGTAACAACGCACC_1 | - | - |
| I21 |  | K1_TAGGCATCATGGGACA_1 | - | - |
| I22 |  | K2_AGCGGTCAGTAACCCT_1 | - | - |
| I26 |  | I2_CAGATCAAGGGCATGT_1 | - | - |
| I27 |  | I5_GTCATTTTCATGCAAC_1 | - | - |
| I29 |  | K1_CACAGGCAGTCCCACG_1 | - | - |
| I30 |  | I4_CAGCCGAGTGTGAAAT_1 | - | - |
| I32 |  | K1_CTAACTTCACCTGGTG_1 | - | - |
| I33 |  | I6_AACGTTGTCCGCAAGC_1 | - | - |
| I34 |  | I3_CAAGGCCTCGTCTGCT_1 | - | - |
| I37 |  | I4_GCAGTTATCCATGCTC_1 | - | - |
| I38 |  | SCoV1-M0_TCTATTGTCAGTACGT_1 | - | - |
| I41 |  | I3_GATCGATAGGAGTACC_1 | - | - |
| I42 |  | I3_TCAGGATCAGTCAGCC_1 | - | - |
| I43 |  | I3_CATGACATCTCCGGTT_1 | - | - |
| I45 |  | K2_GGTGAAGGTGTATGGG_1 | - | - |
| I46 |  | K2_GTGTGCGCAGTGGGAT_1 | - | - |
| I48 |  | I4_CGAGAAGCACGGCGTT_1 | - | - |
| I49 |  | I5_CCTAGCTCAAGGCTCC_1 | - | - |
| I50 |  | I6_AACCATGCACACCGCA_1 | - | - |
| I51 |  | I3_GTTTCTATCCAAATGC_1 | - | - |
| I52 |  | I5_ACACCCTTCCACTCCA_1 | - | - |
| I53 |  | K2_ATTACTCAGATGTCGG_1 | - | - |
| I55 |  | K1_GATGAAAGTGATAAAC_1 | - | - |
| I56 |  | I7_GCAGCCACATTCCTGC_1 | - | - |
| I57 |  | K4_TACCTTAGTTCACCTC_1 | - | - |
| I58 |  | I7_AAACGGGGTACCGAGA_1 | - | - |
| I63 |  | K4_TGGCCAGCAAGGACAC_1 | - | - |
| I64 |  | K4_AGCATACCATGACGGA_1 | - | - |
| I66 |  | I7_TTGGCAAAGTTAGCGG_1 | - | - |
| I67 |  | I8_CCTATTACAATCTGCA_1 | - | - |
| I69 |  | I9_AACCGCGCATCGATTG_1 | - | - |
| I70 |  | I9_CTACCCAAGCCTTGAT_1 | - | - |
| I71 |  | I9_CTGATAGGTTAGTGGG_1 | - | - |
| I72 |  | I9_CAGCTAAGTAACGCGA_1 | - | - |
| I73 |  | I10_CTGCTGTTCCGAAGAG_1 | - | - |
| I78 |  | I9_AGAGCTTTCACTGGGC_1 | - | - |
| I80 |  | I9_CTACCCAGTACCGCTG_1 | - | - |
| I81 |  | I9_TCAGCAATCATTCACT_1 | - | - |
| I82 |  | I9_CCTACCAGTCTACCTC_1 | - | - |
| I83 |  | I9_AGTGGGAGTGCCTTGG_1 | - | - |
| I84 |  | I9_AGTAGTCTCGTACCGG_1 | - | - |
| I85 |  | I10_CATCGGGTCCTAGTGA_1 | - | - |
| I86 |  | I10_TCCCGATTCAGAGCTT_1 | - | - |
| I87 |  | I10_ATCCACCCACGTCTCT_1 | - | - |
| I88 |  | I10_TAAGCGTCAGGTGGAT_1 | - | - |
| I89 |  | SCoV11-M0_CATATTCGTAGGAGTC_1 | - | - |
| I92 |  | I6_CGCTTCAGTGTGGCTC_1 | - | - |
| I94 |  | SCoV1-M0_TCCACACAGGCATGTG_1 | - | - |
| I99 |  | I7_ATGCGATGTCTTTCAT_1 | - | - |
| I100 |  | I10_GGTGAAGCACTGTGTA_1 | - | - |

**Table S14. Benchmarks on the “dark matter” capsid protein discovery task (<30% sequence identity subset, N=43).**

| Method | Acc | Prec | F1 | Recall | TP | FP | FN | TN |
| --- | --- | --- | --- | --- | --- | --- | --- | --- |
| diamond_strict | 0.12 | 1.00 | 0.14 | 0.07 | 3 | 0 | 38 | 2 |
| diamond_tophit | 0.14 | 1.00 | 0.18 | 0.10 | 4 | 0 | 37 | 2 |
| mmseqs_profile_strict | 0.07 | 1.00 | 0.05 | 0.02 | 1 | 0 | 40 | 2 |
| mmseqs_profile_tophit | 0.09 | 1.00 | 0.09 | 0.05 | 2 | 0 | 39 | 2 |
| hhsuite_strict | 0.05 | 0.00 | 0.00 | 0.00 | 0 | 0 | 41 | 2 |
| hhsuite_tophit | 0.12 | 1.00 | 0.14 | 0.07 | 3 | 0 | 38 | 2 |
| foldseek_strict | 0.05 | 0.00 | 0.00 | 0.00 | 0 | 0 | 41 | 2 |
| foldseek_tophit | 0.21 | 1.00 | 0.29 | 0.17 | 7 | 0 | 34 | 2 |
| e2e | 0.63 | 0.93 | 0.77 | 0.66 | 27 | 2 | 14 | 0 |
| **lucavirus** | **0.95** | **0.95** | **0.98** | **1.00** | **41** | **2** | **0** | **0** |
| esm | 0.67 | 0.97 | 0.80 | 0.68 | 28 | 1 | 13 | 1 |

**Table S15. Benchmarks on the “dark matter” capsid protein discovery task (<20% sequence identity subset, N=12).**

| Method | Acc | Prec | F1 | Recall | TP | FP | FN | TN |
| --- | --- | --- | --- | --- | --- | --- | --- | --- |
| diamond_strict | 0.08 | 1.00 | 0.15 | 0.08 | 1 | 0 | 11 | 0 |
| diamond_tophit | 0.08 | 1.00 | 0.15 | 0.08 | 1 | 0 | 11 | 0 |
| mmseqs_profile_strict | 0.08 | 1.00 | 0.15 | 0.08 | 1 | 0 | 11 | 0 |
| mmseqs_profile_tophit | 0.08 | 1.00 | 0.15 | 0.08 | 1 | 0 | 11 | 0 |
| hhsuite_strict | 0.00 | 0.00 | 0.00 | 0.00 | 0 | 0 | 12 | 0 |
| hhsuite_tophit | 0.00 | 0.00 | 0.00 | 0.00 | 0 | 0 | 12 | 0 |
| foldseek_strict | 0.00 | 0.00 | 0.00 | 0.00 | 0 | 0 | 12 | 0 |
| foldseek_tophit | 0.08 | 1.00 | 0.15 | 0.08 | 1 | 0 | 11 | 0 |
| e2e | 0.67 | 1.00 | 0.80 | 0.67 | 8 | 0 | 4 | 0 |
| **lucavirus** | **1.00** | **1.00** | **1.00** | **1.00** | **12** | **0** | **0** | **0** |
| esm | 0.58 | 1.00 | 0.74 | 0.58 | 7 | 0 | 5 | 0 |
